# Evaluating the performance of ReaxFF potentials for sp^2^ carbon systems (graphene, carbon nanotubes, fullerenes) and a new ReaxFF potential

**DOI:** 10.3389/fchem.2022.951261

**Published:** 2022-08-29

**Authors:** Zacharias G. Fthenakis, Ioannis D. Petsalakis, Valentina Tozzini, Nektarios N. Lathiotakis

**Affiliations:** ^1^ Istituto Nanoscienze-CNR, Pisa, Italy; ^2^ Theoretical and Physical Chemistry Institute, National Hellenic Research Foundation, Athens, Greece; ^3^ Department of Surveying and Geoinformatics Engineering, University of West Attica, Athens, Greece; ^4^ Department of Marine Engineering, University of West Attica, Athens, Greece; ^5^ NEST, Scuola Normale Superiore, Pisa, Italy

**Keywords:** ReaxFF, graphene, nanotubes, fullerenes, energetics, mechanical properties, phonon band structure

## Abstract

We study the performance of eleven reactive force fields (ReaxFF), which can be used to study sp^2^ carbon systems. Among them a new hybrid ReaxFF is proposed combining two others and introducing two different types of C atoms. The advantages of that potential are discussed. We analyze the behavior of ReaxFFs with respect to 1) the structural and mechanical properties of graphene, its response to strain and phonon dispersion relation; 2) the energetics of (*n*, 0) and (*n*, *n*) carbon nanotubes (CNTs), their mechanical properties and response to strain up to fracture; 3) the energetics of the icosahedral C_60_ fullerene and the 40 C_40_ fullerene isomers. Seven of them provide not very realistic predictions for graphene, which made us focusing on the remaining, which provide reasonable results for 1) the structure, energy and phonon band structure of graphene, 2) the energetics of CNTs versus their diameter and 3) the energy of C_60_ and the trend of the energy of the C_40_ fullerene isomers versus their pentagon adjacencies, in accordance with density functional theory (DFT) calculations and/or experimental data. Moreover, the predicted fracture strain, ultimate tensile strength and strain values of CNTs are inside the range of experimental values, although overestimated with respect to DFT. However, they underestimate the Young’s modulus, overestimate the Poisson’s ratio of both graphene and CNTs and they display anomalous behavior of the stress - strain and Poisson’s ratio - strain curves, whose origin needs further investigation.

## 1 Introduction

The isolation of graphene, 2 decades ago (Novoselov et al. (2004)) provided a new material with extremely interesting and unique properties, both from the scientific and the technological point of view. Graphene is not only the first two-dimensional (2D) material, but it also has unique properties including (among others) the linear bands at the Fermi level (Castro Neto et al. (2009)), the large Young’s modulus (Fthenakis and Lathiotakis (2015)) and thermal conductivity (Berber et al. (2000); Fthenakis and Tománek (2012); Fthenakis et al. (2014)). Moreover, some of those properties are shared with several three-fold coordinated carbon structures, which can be considered as graphene derivatives, e.g. carbon nanotubes (Iijima (1991)), fullerenes (Kroto et al. (1985)), haeckelites (pentaheptites (Terrones et al. (2000); Fthenakis and Lathiotakis (2015); Crespi et al. (1996)), tetraoctites (Liu et al. (2012); Sheng et al. (2012); Fthenakis and Lathiotakis (2015)), or even three-dimensional structures (Côté et al. (1998); Liu and Cohen (1992); Fthenakis (2016)), foams (Zhu et al. (2014); Inagaki et al. (2015); Liu et al. (2020); Bellucci and Tozzini (2020)), honeycombs (Fthenakis (2017); Krainyukova and Zubarev (2016)), which also attracted a lot of interest.

Several theoretical and computational studies have been devoted to those systems (Bellucci and Tozzini (2020); Berber et al. (2000); Castro Neto et al. (2009); Côté et al. (1998); Crespi et al. (1996); Fthenakis (2016, 2017); Fthenakis and Lathiotakis (2015); Fthenakis and Tománek (2012); Fthenakis et al. (2014); Liu and Cohen (1992); Liu et al. (2012); Terrones et al. (2000); Zhu et al. (2014)). Depending on the size, more or less accurate methods are appropriate. For relatively small systems (up to hundreds of atoms^1^), the more accurate, but more computationally demanding *ab initio* methods, can be used. For larger systems, however, these become practically unfeasible to be performed and therefore less time consuming methods should be used. Semi-empirical methods using parameterized approximations for the superposition integrals (Elstner et al. (1998); Lathiotakis et al. (1996); Fthenakis et al. (2003); Hoffmann (1963)) can apply up to thousands of atoms. For even larger systems, one must abandon the explicit quantum description of electronic structure and adopt classical potentials, also called “Force Fields” (FF), implicitly including the effect of electrons. These potentials are usually analytic expressions of the energy of the system as a function of the internal coordinates, with parameters fitted onto *ab initio* calculations or based on experimental data of different origin (Schuler et al. (2001); Weiner et al. (1984, 1986)). Since the parameterization is typically optimized based on a given set of configurations and/or in given chemical-physical conditions, its interpolation or extrapolation in different situations implies possible inaccuracies raising the well-known problem of the FF transferability.

Historically, the first attempt to develop a classical potential for carbon lead to the Tersoff potential (Tersoff (1988)). Its analytical form (Abell (1985)) describes the dependence of the interaction on the bond order (BO), allowing the treatment of different carbon allotropes (Berber et al. (2000); Fthenakis and Tománek (2012); Fthenakis et al. (2014); Zhao et al. (2019); Ng et al. (2013)). Similar potentials were subsequently proposed, by Chelikowsky (Chelikowsky (1992)), Khor–Das Sharma (Khor and Das Sarma (1988)) and Takai *et al* (Takai et al. (1990)). Moreover, different parameterizations of the original Tersoff potential were developed attempting to provide a more accurate description of phonon dispersion relation (Lindsay and Broido (2010)) and mechanical properties (Rajasekaran et al. (2016)) in graphene and diamond (Shi et al. (2021)). Subsequently, an new version of the Tersoff potential was suggested by Brenner (Brenner (1990)) to extend the FF to hydrocarbons, improve the description of conjugation and sp^2^ and sp^3^ bonds.

The potentials described so far, may be considered as the first generation of BO potentials (BOPs). The second generation includes the so called “Reactive Empirical Bond Order” (REBO) potentials REBO-I (Brenner et al. (2002)) and REBO-II (Pastewka et al. (2008)). In these, the BO is described in terms of *σ* − *π* and *π* contributions, allowing the description of covalent intramolecular bonding, breaking and formation, and including dihedral angle torsional interactions. Several such potentials have been developed to represent elements beside carbon, e.g. S and H (Beardmore and Smith (1996); Dyson and Smith (1996)), or O and H (Ni et al. (2004); Fonseca et al. (2011)) or F and H (Jang and Sinnott (2004)). One issue of these potentials is the poor representation of the van der Waals (vdW) forces, preventing the description of inter-molecular interactions. The so called “Long range Carbon Bond Order Potentials” (LCBOPs) were therefore developed, available in two subsequently improved versions LCBOP-I (Los and Fasolino (2003)) and LCBOP-II (Los et al. (2005)). With the same aim, the so called “Adaptive Intermolecular Reactive Empirical Bond Order (AIREBO)” potentials (Stuart et al. (2000)) and AIREBO(-M) (O’Connor et al. (2015)) were developed.

A different approach is adopted in the class of molecular mechanics FFs (MM-FFs), characterized by a distinction between bonded and non bonded terms of the interactions, implying that the chemical connectivity, or topology, must be given as an input in the model. As a consequence, the energy can be written as a sum of energy contributions from bond stretching, bond angle bending, proper and improper dihedral angle torsion terms, i.e., the chemical or bonding terms assigned based on the given topology, plus terms describing van der Waals interactions and electrostatics. With respect to BOPs and reactive FFs, these are much simpler concerning the analytical forms and implementation, more numerically robust and, up to two orders of magnitude computationally cheaper. On the other hand, they are a step lower in the scale of transferability, requiring different sets of parameters optimized for different cases. For this reason, many different such FF have been developed. A non exhaustive list includes: MM2 (Allinger (1977)), MM3 (Allinger et al. (1989)) and MM4 (Allinger et al. (1996)) for hydrocarbons, the universal FFs UFF (Rappe et al. (1992)) and COMPASS (Sun (1998)), and those specialized for bio-molecules, such as Amber (Weiner et al. (1984; 1986)), CHARMM (Brooks et al. (1983); Nilsson and Karplus (1986)), DREIDING (Mayo et al. (1990)), GROMOS (Schuler et al. (2001)). A potential of that category has been also developed by Fthenakis *et al.* (Fthenakis et al. (2017); Chatzidakis et al. (2018); Kalosakas et al. (2013, 2021)) for the description of three-fold coordinated carbon systems. The main drawback of these FFs is their inability to describe chemical reactions.

Aiming to fix this problem, the group of Van Duin provided new ideas resulting in the ReaxFF potentials (van Duin et al. (2001)), which can be considered as an evolution of the BOPs and are now the state-of-the-art potentials to reproduce reactivity. Indeed, their (large number of) parameters are fitted to a large training set of atomic arrangements in the configuration space provided by *ab initio* simulations, which include also reactive chemical conditions. To the best of our knowledge, the FFs belonging to the ReaxFF class developed for C and other elements are:

1) RDX (Strachan et al. (2003)), originally developed to study the chemistry of nitramine explosions
2) CHO-2008 (Chenoweth et al. (2008)), for the combustion of hydrocarbons
3) Budzien potential (Budzien et al. (2009)), including interactions between C, N, O and H
4) Mattsson potential (Mattsson et al. (2010)), including interactions between C, O and H
5) CHON-2010 (Kamat et al. (2010)), developed to study the formation of soot particles and their interactions with several substances including noble gases
6) The low gradient (lg) potential (Liu et al. (2011)), an extension of the RDX potential including London dispersion terms
7) The charge-implicit ci-CH (Kański et al. (2018)) potential for hydrocarbons improved in terms of computational cost
8) C-2013 (Srinivasan et al. (2015)) for carbon condensed phases
9) CHO-2016 (Ashraf and van Duin (2017))–CHON-2019 (Kowalik et al. (2019)), subsequent improvements of CHO-2008 including C parameters from C-2013 and parameters for N, for simulations of (bio) polymers

Although (on general grounds) ReaxFFs are more accurate than BOPs and more general than MM-FFs, the earlier ReaxFF potentials still suffered from transferability problems, being trained on specific systems and under specific conditions. The subsequent evolution brings improvements in this respect: C-2013 was developed to replace CHO-2008 for the study of carbon condensed phases (Jensen et al. (2015); Srinivasan et al. (2015)), while CHO-2016 was aimed at improving the performances on small hydrocarbons (Ashraf and van Duin (2017)).

Here, we test the performance of the above mentioned ReaxFFs, i.e. potentials (1)-(9) and GR-RDX-2021, on graphene and other sp^2^ carbon systems (nanotubes and fullerenes) as a first step for the evaluation of their adequacy to be used in the study of interactions between these systems and other molecules, which is the focus of a forthcoming paper. In detail, we study: 1) the structural, energetic and mechanical properties of graphene, its response to strain and phonon dispersion, 2) the structural, energetic and mechanical properties of (*n*, 0) and (*n*, *n*) carbon nanotubes (CNTs), and their response to strain up to the fracture limit and 3) the structural and energetic properties of the 40 C_40_ fullerene isomers and the icosahedral C_60_, examining the predictions of the considered potentials with respect to the pentagon adjacency penalty rule. Seven of those potentials predict a Poisson’s ratio value near to unit. Such an unphysical value affect significantly the mechanical or thermal deformations of graphene. We therefore focus on the remaining potentials, namely, C-2013, CHO-2016/CHON-2109 and we propose a new one, called GR-RDX-2021, which is a combination of the C-2013 and RDX potentials with improved accuracy obtained by a limited reintroduction of the concept of atom type.

## 2 Methods

### 2.1 Model systems and general setup

**FIGURE 1:**
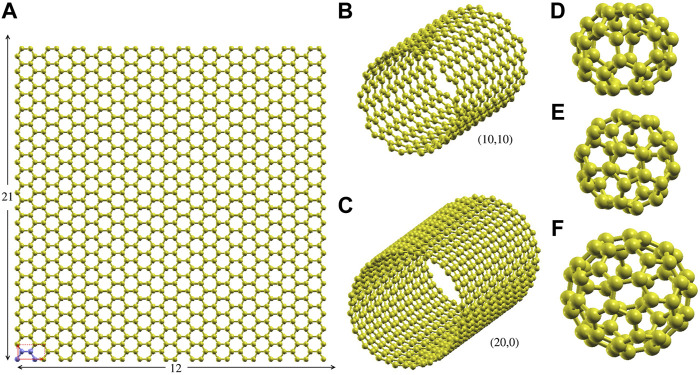
Supercells and representative structures. **(A)** The 1,008 atom 12 × 21 graphene rectangular supercell (size ≈51.12 Å × 51.65 Å). The 4-atom rectangular unit cell is shown in the left down corner of the structure (red rectangle). **(B)** and **(C)** The supercell of the (10.10) and (20.0) carbon nanotubes, as representatives of the (*n*, *n*) and (*n*, 0) nanotubes. **(D)**, **(E)** The no. 38 and 39 C_40_ fullerene isomers, as representatives of the 40 C_40_ fullerene isomers. **(F)** The icosahedral C_60_ fullerene.

Simulations and calculations were performed with the software LAMMPS (Thompson et al. (2022)). Periodic boundary conditions were applied using the natural periodicity in xy direction for graphene, and z for nanotubes. In the other directions and for fullerenes, we left gaps of free space of at least 100 Å, to avoid interactions between periodic images. Specifically:

• **graphene**: we used rectangular supercell built by the 12 × 21 repetition of the rectangular 4-atom unit cell along the xy-plane (see Figure 1A, the supercell includes 1,008 atoms).
• **CNTs**: we considered single wall CNTs with (*n*, 0) and (*n*, *n*) chiralities (*n* = 1–100 for (*n*, 0) CNTs and *n* = 1–20 for (*n*, *n*)), at different values of the strain along the axis. The supercells were built by repeating 10 4*n*-atom unit cells.
• **Fullerenes**: we focused on the icosahedral C_60_ and the 40 C_40_ isomers, corresponding to all possible arrangements of pentagonal and hexagonal rings (Fowler and Manolopoulos (1995)).

To calculate the response to strain, structure relaxations are performed at fixed strain using a grid of equidistant strain values along *x* and *y* directions for graphene (arm chair and zig-zag) and along *z* for CNTs. The convergence criteria were set at 10^–6^ kcal/(mole Å) ( $\approx 4\times 10^{-8}$ eV/Å) for the forces on atoms and 10^–4^ kcal/mol (or 4 × 10^–9^ eV/atom) for the global energy variation. The Young’s modulus *E* and the Poisson’s ratio *ν* are calculated using the dependence of energy on strain and correlation between strain components. Phonon dispersion relations in graphene are calculated evaluating the forces for given structural distortions (“frozen phonon” method), as described in the corresponding sections, where additional details of the calculations are reported.

### 2.2 A new hybrid ReaxFF

As it will be shown in the next sections, most of the ReaxFF potentials predict an unrealistic value of *ν*. The solution to this problem might follow the strategy of the generalization of ReaxFFs by training on extended systems, as for the recently developed CHON-2019 potential (Kowalik et al. (2019)), which requires, however, a dedicated effort. In this work, we obtain similar performances in terms of both mechanical properties and reactivity towards organic molecules with a simpler approach based on the combination of previous ReaxFF potentials without reparameterizing them. Specifically we combined C-2013 (Srinivasan et al. (2015)), for the interactions between three-fold coordinated carbons in graphene, with RDX (Strachan et al. (2003)), for all other interactions, within a hybrid new potential that we call *GR-RDX-2021*. This will incorporate the good mechanical properties of C-2013, developed for extended sp^2^ systems, and of RDX developed to describe interactions between C, H, N and O.

To follow this idea, 2 C atoms types are defined: those belonging to graphene (or other three-fold coordinated carbon systems) and all others. Denoting the former as “C_*g*_” and the latter as “C”, one has three kinds of carbon-carbon interactions, namely C_*g*_-C_*g*_, C_*g*_-C and C-C, and three kinds of interaction with other elements (E), namely C_*g*_-E, C-E and E-E. In GR-RDX-2021, C_*g*_-C_*g*_ are described by C-2013, and C-C, C-E, E-E by RDX. C_*g*_-C and C-C interactions are equivalent, as well as, C_*g*_-E and C-E.

ReaxFF potentials have two groups of parameters. The first includes some general ones, e.g. those related to the description of the switch function, same for all interactions. The second group contains those describing the element dependent 2-, 3- and 4-body interactions. As C-2013 and RDX have different parameters of the first group, we chose those from the RDX for the hybrid potential. This means that for interactions of C, H, O, N, and of them with C_*g*_, it behaves as in RDX, while for the C_*g*_-C_*g*_ behaves as a modified C-2013. The general performance of the new potential specifically regarding C_*g*_-C and C_*g*_-E interactions must also be tested, which is the matter of a forthcoming paper. It is worth noting that GR-RDX-2021 is intrinsically capable of representing the sp^2^-sp^3^ and sp^3^ interactions, inherited by C-2013 and can therefore be used to represent carbon foams, nanoporous carbon or diamond-like systems.

The definition of multiple atom types for carbon brings a disadvantage: if C_*g*_ and C atoms interchange their position, the description might be less accurate. However, because C_*g*_-C and C-C interactions are the same, the description is still correct when a C_*g*_ atom takes the place of a C atom. Of course the GR-RDX-2021 potential, carries inaccuracies inherent to both RDX and C-2013, as well as possible ones due to the modifications in C-2013 general parameters. The file with the parameters of GR-RDX-2021 in LAMMPS format is included as the Supplementary Information to this work.

## 3 Results

### 3.1 Graphene

#### 3.1.1 Structural properties and energetics

**FIGURE 2:**
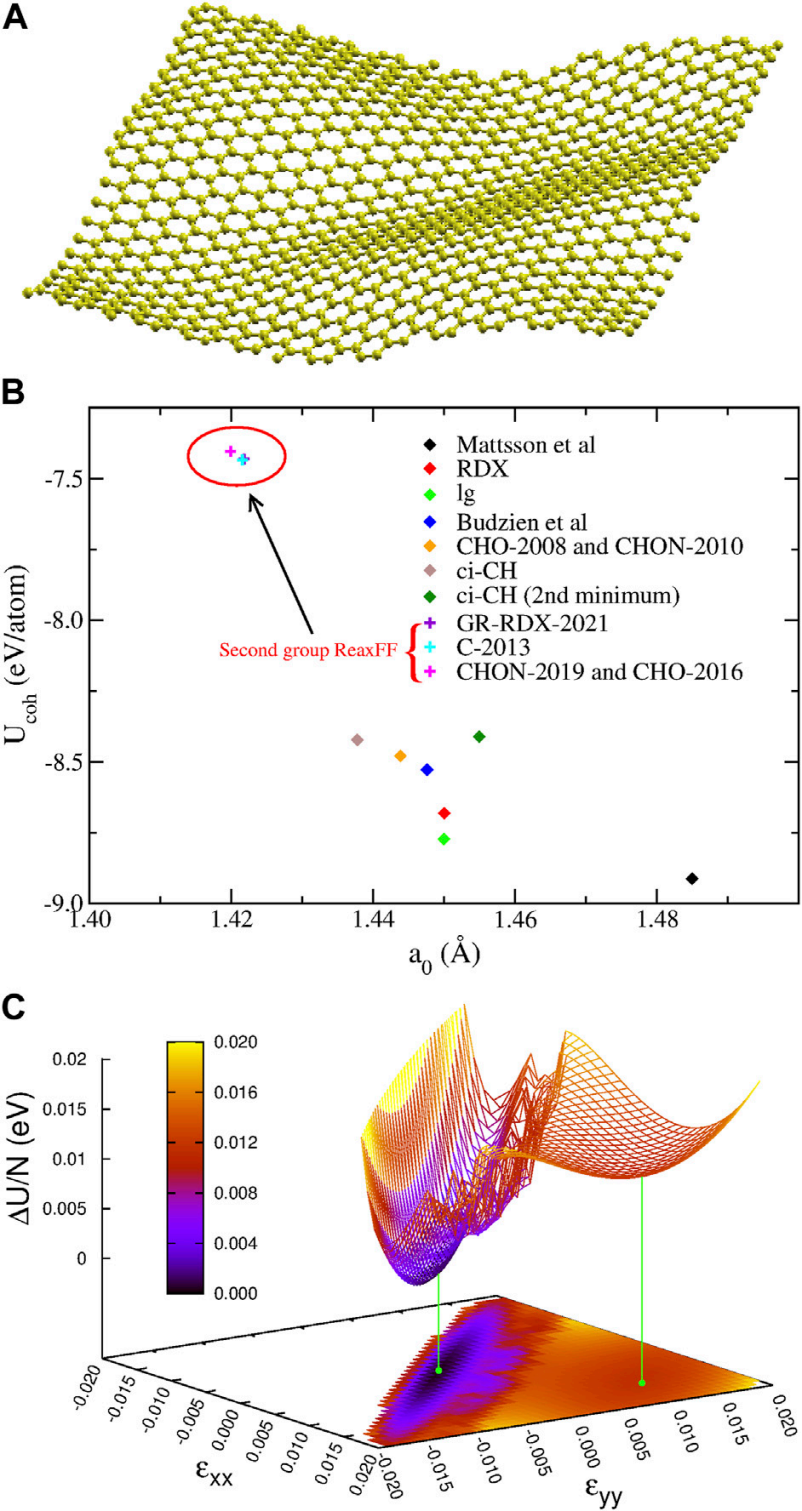
Optimizations for graphene: **(A)** The optimized 1,008 atom 12 × 21 rectangular graphene supercell for *a*_0_ = 1.42 Å using the CHON-2010 potential. **(B)** Cohesive energy *U*_*coh*_ as a function of bond length *a*_0_ for graphene for the different ReaxFF. **(C)** The potential energy surface *U*(*ɛ*_*xx*_, *ɛ*_*yy*_) of graphene derived from ci-CH potential. The two minima are shown with the green points and lines.

Here we present our results for the cohesive energy *U*_*coh*_ and the structural properties of relaxed graphene structures, obtained with the potentials (1)-(9) and GR-RDX-2021. We started optimizations both by exactly flat and rippled structures, to avoid introducing biases and optimizing both the structure and the supercell size. We found flat exact hexagonal symmetries for the stable state in all cases, though with different bond lengths, *a*_0_, reported in Table 1 in descending order, together with the cohesive energies *U*_*coh*_. In the same table, we also report experimental and *ab initio* values for comparison. The potentials can be classified in two groups: the first includes the Mattsson, ci-CH, RDX, lg, Budzien, CHO-2008 and CHON-2010 potentials, and has *a*_0_ in the range [1.48.1.44] Å and *U*_*coh*_ in the range [-8.9,-8.4] eV; the second includes the GR-RDX-2021, C-2013, CHO-2016 and CHON-2019 potentials, and *a*_0_ in the range [1.420.1.422] Å and *U*_*coh*_ in the range [-7.43,-7.40] eV. The values of *a*_0_ for CHO-2008 and C-2013 potentials are in agreement with those found by Lebedeva *et al* (Lebedeva et al. (2019)). The results of *U*_*coh*_ versus *a*_0_ are also presented in Figure 2B, where the distinction between the two groups is evident. While the second group reasonably reproduces the experimental values, the first one overestimates *a*_0_ by 1.4–4.2% and *U*_*coh*_ (in absolute value) by 13.5–20.3%. The bond length overestimation in first group is not that large, but if the supercell size is forced to a value corresponding to the experimental length of 1.42 Å, the structure turns to a rippled one, as shown in Figure 2A obtained with CHON-2010. These results, for contracted graphene are in accordance with other DFT results, where similar ripples appear in laterally compressed graphene sheets (Tozzini and Pellegrini (2011); Rossi et al. (2015)).

**TABLE 1:** Predictions of the ReaxFF potentials considered in the present study for the structural, energetic and mechanical properties of graphene. 1) Cohesive energy *U*_*coh*_ and 2) bond length *a*_0_ of the optimum energetically graphene structure, 3) Young’s modulus *E*_*x*_ = *σ*_*xx*_/*ɛ*_*xx*_ and 4) Poisson’s ratio *ν*_*x*_ = −*ɛ*_*yy*_/*ɛ*_*xx*_ for uniaxial strain *ɛ*_*xx*_ along *x*-direction, 5) Young’s modulus *E*_*yy*_ = *σ*_*yy*_/*ɛ*_*yy*_ and 6) Poisson’s ratio *ν*_*y*_ = −*ɛ*_*xx*_/*ɛ*_*yy*_ for uniaxial strain *ɛ*_*yy*_ along *y*-direction, 6) spring constant *k*_*s*_ for bond stretching and 7) *k*_*b*_ for bond angle bending 8) - 10) the elastic constants *c*_11_ = *c*_22_, *c*_12_ = λ* and *c*_66_ = *G* = *μ* (*G* is the shear modulus, and *λ* and *μ* the first and second Lamé’s coefficients, respectively). Directions *x* and *y* correspond to the arm chair and zig-zag directions, respectively. Ab initio and experimental values are included for comparison. The *k*_*s*_, *k*_*b*_, *c*_11_, *c*_12_ and *c*_66_ values in parenthesis, have been calculated by us, based on the average of the provided *E* and *ν* values. [1]: (Lynch and Drickamer (1966)), [2]: (Lee et al. (2008)), [3]: graphite (Bosak et al. (2007)), [4]: graphite (Blakslee et al. (1970)), [5]: AIMPRO (Ivanovskaya et al. (2010)), [6]: Siesta (Fthenakis and Menon (2019)), [7]: Quantum Espresso and QM-CPACK (Shin et al. (2014)), [8]: Quantum Espresso (Fthenakis and Lathiotakis (2015; 2017)), [9]: (Jensen et al. (2015)) [10]: (Lebedeva et al. (2019)) [11]: (Qian et al. (2021)), * 2nd minimum, ** ±150, *** ±20.

ReaxFF or method	*U*_*coh*_ (eV/atom)	*a*_0_ (Å)	*E*_*x*_/ *E*_*y*_(GPa)	*ν*_*x*_/*ν*_*y*_	*c*_11_ (GPa)	*c*_12_ (GPa)	*c*_66_ (GPa)	*k*_*s*_ (eV/Å^2^)	*k*_*b*_ (eV/;Å^2^)
Mattsson	−8.912227	1.48495	1014/1016	0.987/0.978	38752	38241	255	2884	1.54
RDX	−8.681633	1.45003	1051/1048	0.984/0.984	33102	32573	264	2375	1.60
lg	−8.773100	1.44998	1087/1084	0.983/0.984	33698	33151	273	2417	1.66
Budzien	−8.527977	1.44761	1060/1056	0.984/0.986	35407	34874	267	2541	1.61
CHO-2008	−8.479561	1.44385	1331/1334	0.983/0.983	40234	39562	336	2885	2.03
CHON-2010	−8.479561	1.44385	1331/1334	0.983/0.983	40234	39562	336	2885	2.03
ci-CH	−8.423060	1.43777	926/936	0.975/0.976	19006	18534	236	1357	1.43
ci-CH*	−8.411486	1.45497	821/819	0.753/0.746	1869	1400	234	118	1.52
GR-RDX-2021	−7.431757	1.42183	795/797	0.550/0.550	1141	628	257	64.0	1.81
C-2013	−7.434825	1.42159	801/795	0.537/0.540	1124	605	259	62.5	1.84
CHO-2016	−7.404626	1.41991	765/772	0.543/0.554	1099	603	248	61.5	1.75
CHON-2019	−7.404626	1.41991	765/772	0.543/0.554	1099	603	248	61.5	1.75
exper. [1]		1.4210							
exper. [2]			1020**						
exper. [3]			(1092)	(0.125)	1109	139	485	(45.1)	(4.78)
exper. [4]			(1030)	(0.17)	1060***	180***	(440)	(44.8)	(4.1)
DFT/PBE [5]	−7.73	1.429							
DFT/PBE [6]		1.4372	964/964	0.189/0.190	(1000)	(189)	(405)	(43.0)	(3.70)
DFT/PBE [7]	−7.906								
QMC [7]	−7.464(10)								
DFT/PBE [8]			1024/1020	0.177/0.173	1054	185	435	44.8	4.04
DFT/PBE-D2 [9]		(1.424)	1046	0.139	1067	148	459	(43.9)	(4.45)
CHO-2008 [10]		1.4438496	1343	0.987	(51992)	(51316)	(338)	(3735)	(2.04)
CHO-2008 [9]		(1.421)	1235	0.876	5320	4662	311	(360)	(2.05)
C-2013 [10]		1.4215522	789.4	0.537	(1109)	(596)	(257)	(61.6)	(1.82)
C-2013 [9]		(1.421)	751	0.502	(1004)	(504)	(250)	(54.5)	(1.81)
C-2013 [11]			920		650				

Additionally, ci-CH potential was found to have two minima differing by 0.0116 eV/atom, whose structure differs only by the bond length (of 1.2%), the shorter one being more stable. In order to better understand the origin of this splitting, we calculated the potential energy surface (PES) as a function of the strains *ɛ*_*xx*_, *ɛ*_*yy*_ along the *x* and *y* directions, respectively, shown in Figure 2C. The saddle point connecting the two minima appears at *ɛ*_*xx*_ = *ɛ*_*yy*_ = 0.006, and lies 0.0134 eV/atom above the absolute minimum and 0.0018 eV/atom above the secondary one, which is very shallow. Moreover, the transition region between the minima appears rather noisy, possibly due to reasons described below, in Section 3.1.3).

#### 3.1.2 Mechanical properties

**FIGURE 3:**
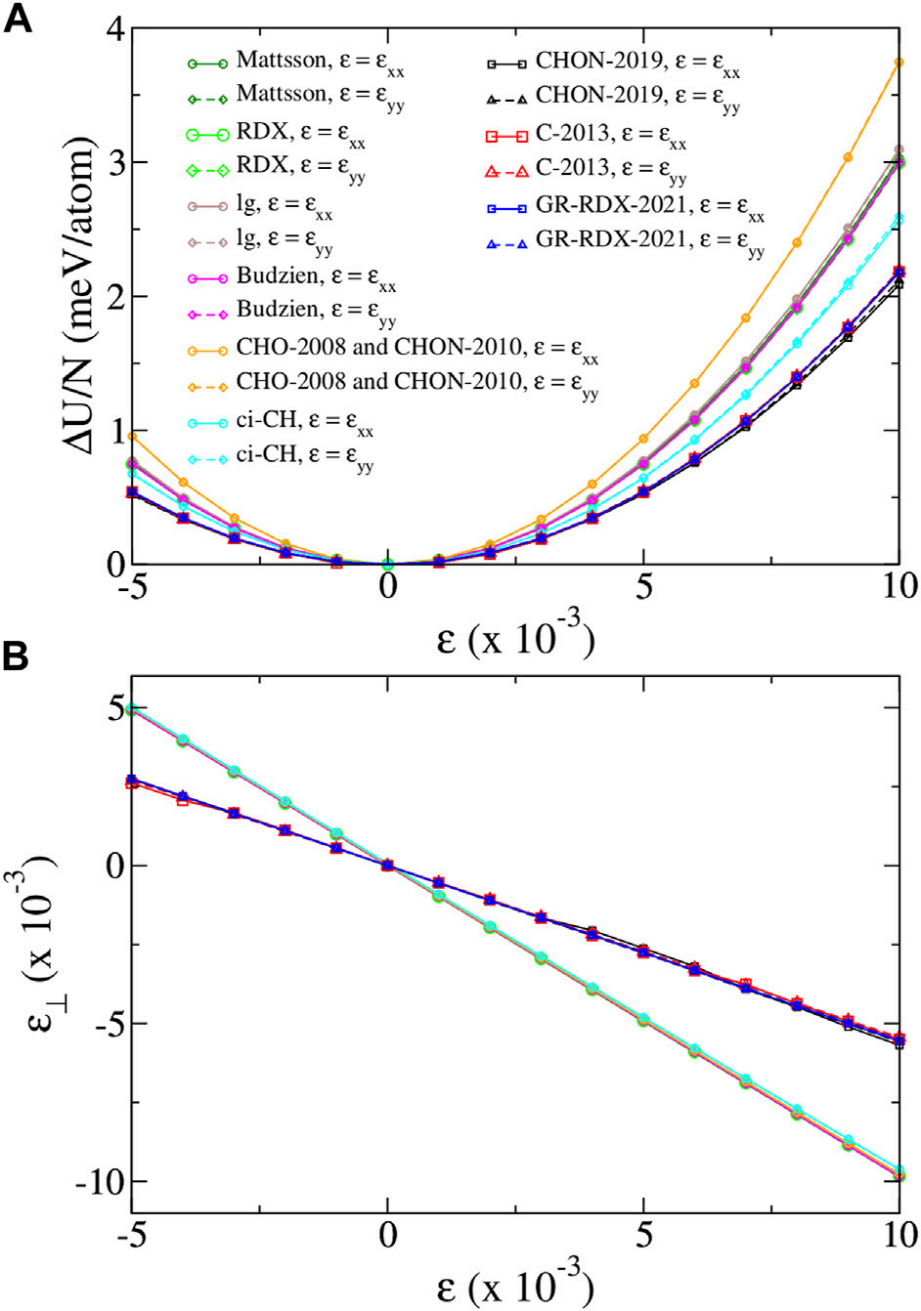
**(A)** Strain energy per atom Δ*U*/*N* and **(B)** transverse strain *ɛ*_⊥_, versus strain *ɛ*, for uniaxial strain along *x* and *y* directions, for the ReaxFF potentials of the present study. For strain along *x* (arm chair) direction, *ɛ* = *ɛ*_*xx*_, *ɛ*_⊥_ = *ɛ*_*yy*_ and *σ*_*yy*_ = 0. For strain along *y* (zig-zag) direction, *ɛ* = *ɛ*_*yy*_, *ɛ*_⊥_ = *ɛ*_*xx*_ and *σ*_*xx*_ = 0. Legends of panel **(A)** apply in both panels.

We calculate the Young’s modulus *E*, and the Poisson’s ratio *ν* for uniaxial strain along *x* and *y* directions and, based on them, the elastic constants *c*_11_, *c*_12_ and *c*_66_. Figure 3A shows the strain energy per atom Δ*U*/*N* of the fully optimized uniaxially strained graphene for low strain values (−0.005 ≤ *ɛ* ≤ 0.01). Here Δ*U* = *U*(*ɛ*) − *U*_0_, with *U*_0_ the energy at zero strain. Figure 3B shows the corresponding transverse relaxed strain *ɛ*_⊥_ vs. the longitudinal imposed one. For small strain values, the energy and the transverse strain has a quadratic and a linear dependence on strain, respectively, as shown in Figures 3A,B. We may then write

$$U\left(\varepsilon_{xx}\right)=\kappa_{x}\varepsilon_{xx}^{2}+U_{0}$$ (1)

$$\varepsilon_{⊥}=\varepsilon_{yy}=-\nu_{x}\varepsilon_{xx}$$ (2)

where *ν*_*x*_ is the Poisson’s ratio for strain along *x* direction. It is easy to show that

$$E_{x}=\sigma_{xx}/\varepsilon_{xx}=2\kappa_{x}/V,\;V=L_{x}L_{y}d_{0},$$ (3)

with *E*_*x*_ being the Young’s modulus for strain along *x* and *V* the volume evaluated using the xy supercell size and the sheet thickness *d*_0_ = 3.34 Å, corresponding to the graphite interlayer separation distance (Fthenakis and Lathiotakis (2015)). The corresponding equations along *y* direction are obtained by interchanging x with y, i.e., $U\left(\varepsilon_{yy}\right)=\kappa_{y}\varepsilon_{yy}^{2}+U_{0}$ , *ɛ*_⊥_ = *ɛ*_*xx*_ = −*ν*_*y*_ *ɛ*_*yy*_, and *E*_*y*_ = *σ*_*yy*_/*ɛ*_*yy*_ = 2*κ*_*y*_/*V*. The values of *κ*_*x*_ and *κ*_*y*_ (and of *E*_*x*_ and *E*_*y*_) can be found with a quadratic fit of the (*ɛ*, Δ*U*/*N*) points of Figure 3A, while *ν*_*x*_ and *ν*_*y*_ with a linear fit to (*ɛ*, *ɛ*_⊥_) of Figure 3B. The *E*_*x*_, *E*_*y*_, *ν*_*x*_ and *ν*_*y*_ values found here for the ReaxFF potentials are reported in Table 1, and compared with the DFT theoretical predictions and experimental values, as well as, with the results found by Qian *et al* (Qian et al. (2021)) for C-2013 potential, and by Jensen *et al* (Jensen et al. (2015)) and Lebedeva *et al* (Lebedeva et al. (2019)) for both C-2013 and CHO-2008 potentials.

We first observe that Figure 3 shows basically no anisotropy: all curves depending on *ɛ*_*xx*_ superimpose to the corresponding for *ɛ*_*yy*_, leading to (almost) identical values of the corresponding elastic moduli. Figure 3B also clearly shows that all data collapse on two lines only, corresponding to potentials of the two groups previously defined according to the cohesive energy predictions. The first group returns an unrealistically high *ν* ∼ 0.983–0.987, while the second returns values 0.537–0.554 nearer to the DFT one, although still substantially overestimated. In turn, the curves of panel 1) for the potentials of the first group are very similar, yielding *E* ∼926–1,087 GPa, while for the potentials of the second group they collapse to the same curve yielding *E* ∼ 765–801 GPa. The results we find for the CHO-2008 potential, are very similar with those found by Lebedeva et at (Lebedeva et al. (2019)), although not very similar with those found by Jensen *et al* (Jensen et al. (2015)). Moreover, the results we find for the C-2013 potential are very similar with those found by both Lebedeva *et al* (Lebedeva et al. (2019)) and Jensen *et al* (Jensen et al. (2015)), though slightly different from those of Qian *et al* (Qian et al. (2021)). Therefore, on average, *E* values from first group matches better experimental and DFT data than that by the second, which yields a value lower by at least 25%. This could have been considered as a success of the first-group potentials, if their *ν* values were not so large, meaning that deformations would be practically achieved only through bond angle bending, with negligible bond stretching.

Table 1 also reports the elastic constants, related to *ν* and *E* (see Ref. Chou and Pagano (1992))

$$c_{11}=c_{22}=\frac{E}{1-\nu^{2}},$$ (4)

$$c_{12}=\lambda^{*}=\frac{E\nu }{1-\nu^{2}},$$ (5)

$$c_{66}=G=\mu =\frac{E}{2\left(1+\nu \right)},$$ (6)

with these equations being valid for isotropic 2D materials. *G* is the shear modulus, coinciding with the second Lamé’s coefficient *μ*, and λ* is the first Lamé’s coefficients for 2D isotropic materials (differing by its three-dimensional version which is *λ* = *νE*/[(1 + *ν*) (1 − 2*ν*)]). The elastic constants *c*_11_ and *c*_12_ predicted by the potentials of the first group are extremely high due to the 1 − *ν* ^2^ in the denominator of Eqs. 4, 5. For the second group the *c*_11_ values are very close to those derived from DFT, but the *c*_12_ are approximately 3–4 times larger. As for the shear modulus *G* (or the *c*_66_ elastic constant), the first and second group predict more or less the same value, approximately half the value derived by DFT.

To understand the origin of the discrepancies between the ReaxFF potentials and the experimental and DFT results, we evaluate the bond stretching and bending spring constants *k*_*s*_ and *k*_*b*_ of an equivalent stick and spiral model in terms of *E* and *ν*. Within this model (Fthenakis and Lathiotakis (2017)) the deformation energy Δ*U* of graphene is

$$\Delta U=\frac{1}{2}\underset{i}{\sum }\left(k_{s}\delta l_{i}^{2}+\frac{1}{2}\underset{j}{\sum }k_{b}a_{0}^{2}\delta \phi_{ij}^{2}\right),$$ (7)

where *δl*_*i*_ = *l*_*i*_ − *a*_0_ is *i*th bond elongation, *δϕ*_*ij*_ = *ϕ*_*ij*_ − *ϕ*_0_ is the deformation of the angle between bonds *i* and *j* with respect to the sp^2^ angle *ϕ*_0_ = 120^*o*^. Using Eq. 7, one gets

$$E=\frac{8\sqrt{3}}{d_{0}}\frac{k_{s}k_{b}}{k_{s}+18k_{b}}\;and\;\nu =\frac{k_{s}-6k_{b}}{k_{s}+18k_{b}}$$ (8)

or equivalently

$$k_{s}=\sqrt{3}d_{0}\frac{E}{1-\nu }\;and\;k_{b}=\frac{d_{0}}{2\sqrt{3}}\frac{E}{3\nu +1}.$$ (9)

Fitting DFT data (Fthenakis and Lathiotakis (2017)) to this model leads to the values *k*_*s*_ ≈ 45 eV/Å^2^ and *k*_*b*_ ≈ 4 eV/Å^2^, in consistency with *E* = 1,012 GPa and *ν* = 0.1744 also obtained by DFT (Fthenakis and Lathiotakis (2015)). Using the *E* and *ν* values for each potential we derived *k*_*s*_ and *k*_*b*_, shown in the last columns of Table 1. As previously, the spring constants are different for the two potential groups. For the first group, *k*_*s*_ is 1,357–2885 eV/Å^2^ and *k*_*b*_ 1.43–2.03 eV/Å^2^, i.e. the bond stretching is 30–64 times stronger than that provided by DFT, while the bond-angle bending is weaker by a factor ≈1/3−1/2 . The extremely high *k*_*s*_ values are due to near unit value of *ν* in the potentials of the first group. For the second group, *k*_*s*_ ≈ 61.5–64 eV/Å^2^ and *k*_*b*_ ≈ 1.75–1.84 eV/Å^2^, i.e., only ≈4/3 stronger and ≈2/5 weaker than the corresponding DFT values, respectively. As a consequence, the energy penalty e.g. for *ɛ* = 0.01 stretching in the first group, is Δ*U* = 260 meV, while the same amount of energy is sufficient to generate an angular distortion as large as *δϕ* = 23^*o*^, or a stretching of up to 7% with the potentials of the second group or DFT. Conversely, for the second group, the amount of energy needed for a stretching of 1% is only 4.5–6.3 meV. As a summary of the above discussion, and to highlight the importance of the correct reproduction of the Poisson ratio, we observe that from Eq. 9 one gets

$$\frac{k_{s}}{k_{b}}=6\frac{3\nu +1}{1-\nu },$$ (10)

which clearly shows the divergence of the *k*_*s*_/*k*_*b*_ ratio as *ν* → 1. In conclusion, the potentials of the first group might provide unreliable results for graphene. Therefore, in the following we will focus only on those of the second group.

#### 3.1.3 Response to strain

**FIGURE 4:**
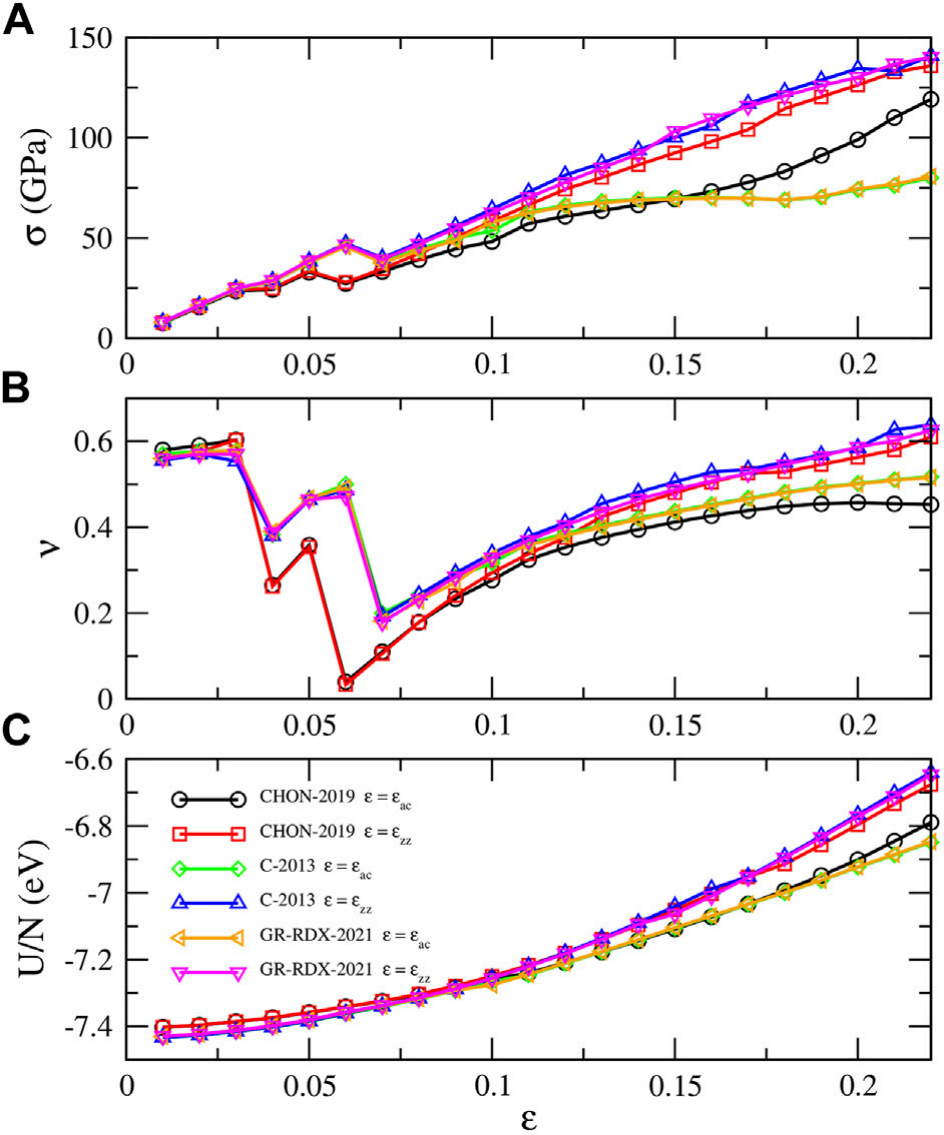
Response of graphene to uniaxial strain along arm chair and zig-zag directions. **(A)** Stress *σ*, **(B)** Poisson’s ratio *ν* and **(C)** energy per atom *U*/*N* along the strain direction. The legends of panel **(C)** applies in all panels.

Figure 4 shows the energy per atom (*U*/*N*), stress (*σ*) and Poisson’s ratio (*ν*) vs*.* *ɛ* of the potentials of the second group, for uniaxial strain along zig-zag and arm chair directions in the range [0, 0.22]. The results by C-2013 and GR-RDX-2021 are similar, and not very different from those by CHON-2019. The behavior is isotropic at low strain values, while anisotropy appears at *ɛ*⪆0.10. The break of isotropy and harmonicity occurs even earlier, at *ɛ* = 0.07 in panel (a), while DFT calculations (Fthenakis and Menon (2019); Fthenakis and Lathiotakis (2015)) show a similar behavior at *ɛ*⪆0.15. ReaxFF agrees with DFT in predicting a smaller stiffness at large stress along the zig-zag with respect to the armchair direction.

Additionally, the stress in Figure 4A shows a net drop occurring at *ɛ* ≈ 0.05–0.06 for the different potentials, also observed in the studies by Jensen *et al* (Jensen et al. (2015)) and by Qian *et al* (Qian et al. (2021)). These “drops” are accompanied by strain “jumps” in the lateral direction, which results in corresponding “jumps” in the Poisson’s ratio (panel B). Similar results for the Poison’s ratio were obtained by Lebedeva *et al* (Lebedeva et al. (2019)) using the C-2013 ReaxFF. While up to *ɛ* ≈ 0.03 the sheet normally “shrinks” in the lateral strain direction at *ɛ* ≈ 0.04 it displays an expansion and a subsequent shrinking, but with different coefficients dependent on the potentials. These drops repeat a couple of time before *ν* assumes a monotonically increasing though non linear behavior. These alternating jumps in *ν* are not observed in DFT studies (Fthenakis and Menon (2019); Fthenakis and Lathiotakis (2015)), where a smoothly decreasing behavior is observed.

**FIGURE 5:**
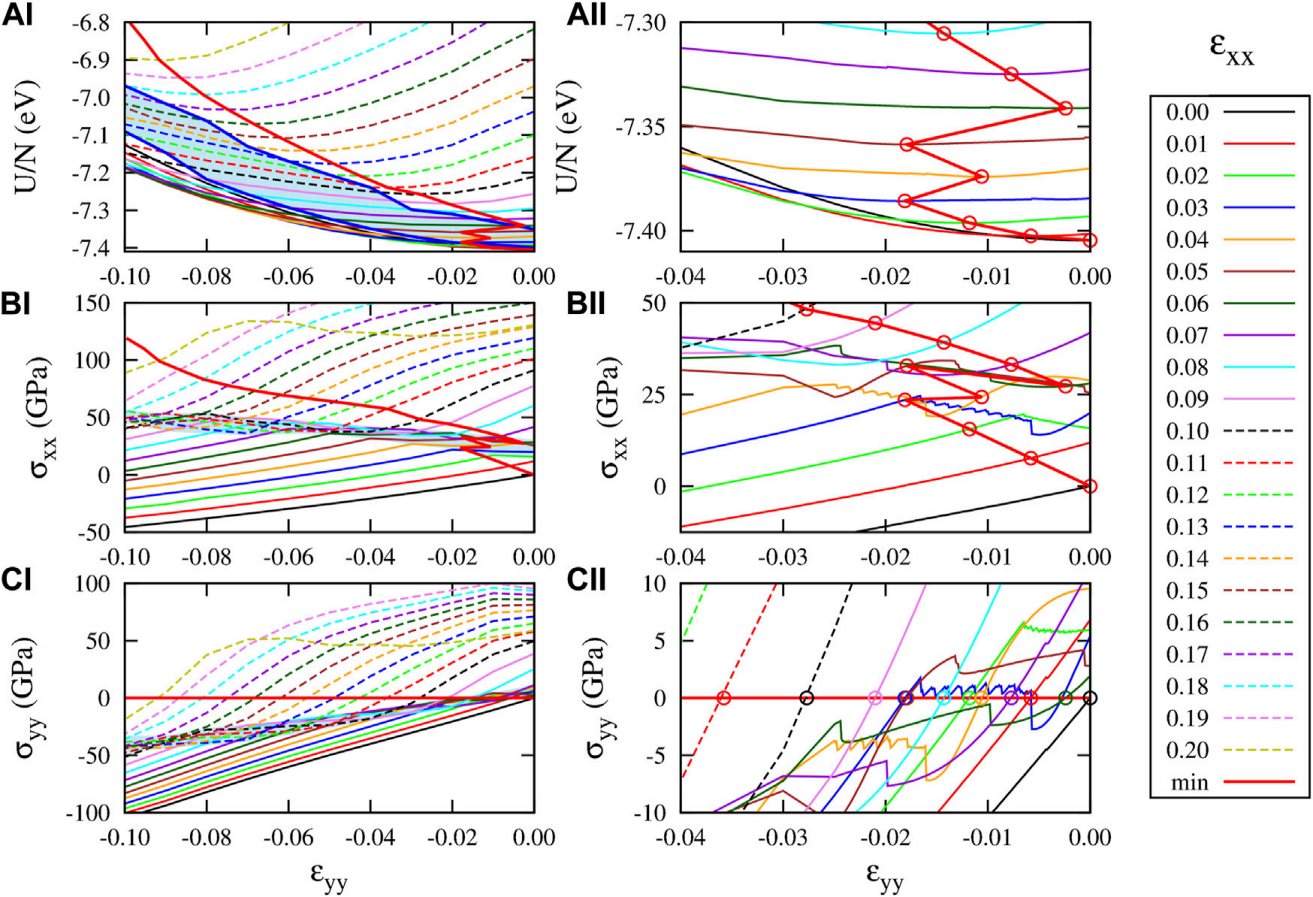
Response of graphene to strain *ɛ*_*xx*_ and *ɛ*_*yy*_ according to the CHON-2019 potential. (AI) And (AII) *U*(*ɛ*_*xx*_, *ɛ*_*yy*_)/*N* (BI) and (BII) *σ*_*xx*_(*ɛ*_*xx*_, *ɛ*_*yy*_), and (CI) and (CII) *σ*_*yy*_(*ɛ*_*xx*_, *ɛ*_*yy*_). The right panels ((AII) (BII) and (CII)), present the same results with those presented in the left panels ((AI) (BI) and (CI)) but in different scale range (−0.04 ≤ *ɛ*_*yy*_ ≤ 0) to provide details for the “jumps” of the optimum strained graphene structure at low strain *ɛ*_*xx*_ values. The curves presented in those plots correspond to constant strain *ɛ*_*xx*_, for the *ɛ*_*xx*_ values shown in the legends. The *ɛ*_*yy*_ increment in these plots is 10^–4^.

To get further insight in this anomalous behavior of *ν*, we performed a finer sampling of the energy and stress values in the *ɛ*_*xx*_- *ɛ*_*yy*_ plane, using CHON-2019, reported as a function of *ɛ*_*yy*_ at given values of *ɛ*_*xx*_ in Figure 5, left side panels. The right side panels reports a zoom into the anomalous region. The thick red solid line crossing the curves in each panel connects the equilibrium points at given constant *ɛ*_*xx*_. The anomalous behavior is visible in (panels B) and (C) of Figure 5 as a narrow shaded strip where the curves change slope. In (panels C) the strip connects approximately the points (*ɛ*_*yy*_, *σ*_*xx*_) ≈ (-0.10, 50 GPa)–(0, 25 GPa), in (panels C) the points (*ɛ*_*yy*_, *σ*_*yy*_) ≈ (-0.10, -40 GPa)–(0, 5 GPa). Below the strip, the system behaves elastically, with almost linear dependence of *σ* on *ɛ*. Above the strip, the behavior appears again almost linear, but with a different slope suddenly changing in the strip area. For instance, the stress curve for *ɛ*_*xx*_ = 0.09 change its slope at *ɛ*_*yy*_ ≈ − 0.03 and *ɛ*_*yy*_ ≈ − 0.08. The corresponding transition area in the energy plots (panels A) has clearly a curve shape less easily identifiable, which can be estimated from the *σ* − *ɛ* plots. In panel (AI) it is shown as the shaded area between the thick blue lines.

Inside the border strips, the system displays on average an opposite dependence of strain on stress, which can be roughly estimated as the slope of the strip itself, namely ≈−250 GPa for *σ*_*xx*_ and ≈450 GPa for *σ*_*yy*_. The negative and positive slopes along *x* and *y* directions, respectively, show the tendency of the PES inside that area to form a saddle point. In general, therefore, moving along the red lines in (panels B) and (C), one crosses the different *ɛ*_*xx*_ lines and can rebuilt plots of Figure 4. Thus, for 0 ≥*ɛ*_*xx*_ ≥ 0.03, the energy minimum falls in the region below that strip, exhibiting a linear-like behavior between stress and strain with a specific slope, as shown in Figure 4A, which is also depicted as a linear relation of *ν* versus strain in Figure 4B for that strain range. For 0.03 < *ɛ*_*xx*_ < 0.07, however, it falls inside that strip area and the slope of *σ*_*xx*_ versus *ɛ*_*xx*_ changes having an irregular (not linear) behavior, which is also depicted in the stress–strain plot of Figure 4A for that strain range. Thus, graphene in the lateral strain direction either enlarges or shrinks also irregularly, causing the irregular behavior of *ν*, which can be seen in Figure 4B for that strain range. For *ɛ*_*xx*_ ≥ 0.07 the energy minimum falls in the region above that strip, exhibiting again an almost linear behavior, as shown by the thick red line of Figure 5B and Figure 4A.

**FIGURE 6:**
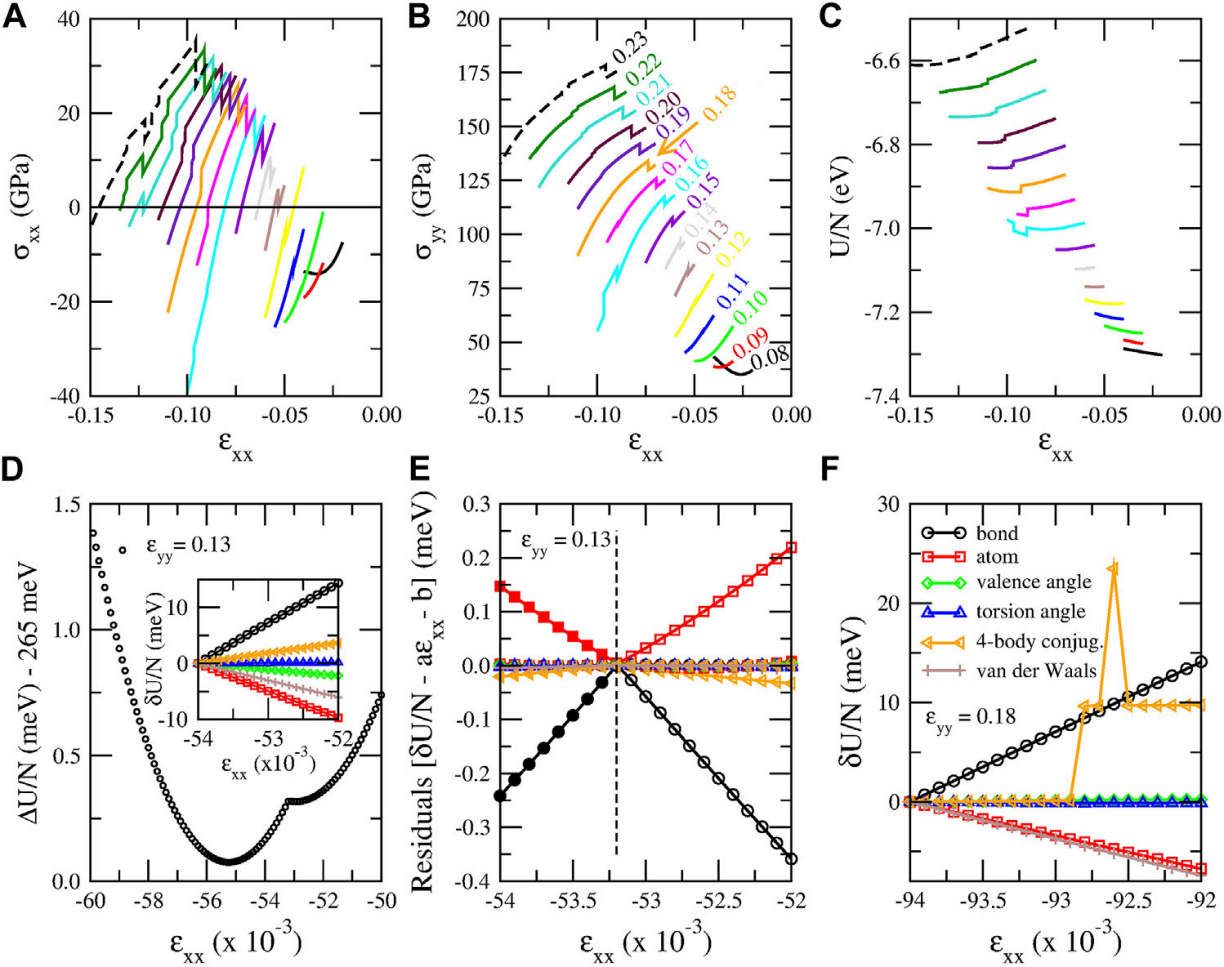
**(A)**–**(C)** “Jumps” (discontinuities) in stress and energy plots obtained using the CHON-2019 potential. **(A)** Stress along *x* direction (*σ*_*xx*_), **(B)** stress along *y* direction (*σ*_*yy*_) and **(C)** energy per atom (*U*/*N*) as a function of strain along *x* direction (*ɛ*_*xx*_) for fixed *ɛ*_*yy*_ values indicated with different colors. The *ɛ*_*yy*_ value corresponding to each curve of panels **(A)**, **(B)** and **(C)** is shown next to each curve in panel **(B)** with the same color. **(D)** Strain energy per atom Δ*U*/*N* = *U*/*N* − *U*_*coh*_ shifted by 265 meV versus *ɛ*_*xx*_ for *ɛ*_*yy*_ = 0.13, showing the discontinuous change in the energy slope. In the inset: energy per atom for the six terms constituting the energy of the system. Each energy term is shifted by the value it has for *ɛ*_*xx*_ = −0.0540. **(E)** Residuals between the energy per atom values of each energy term shown in the inset of panel **(D)** and their fitted linear functions for −0.0540 ≤ *ɛ*_*xx*_ ≤ −0.0532 (open symbols) and for −0.0532 ≤ *ɛ*_*xx*_ ≤ −0.0520 (solid symbols). The residuals are calculated for all the energy values in the [-0.0540, -0.0520] interval and not only for the values used for the fitting.**(F)** The six energy terms for *ɛ*_*yy*_ = 0.18 and −0.0940 ≤ *ɛ*_*xx*_ ≤ −0.0920. The legends of panel **(F)** apply both in panel **(E)** and the inset of panel **(D)**. The *ɛ*_*xx*_ increment in all these plots is 10^–4^.

Beyond that anomalous behavior of graphene PES, panels (BII) and (CII) of Figure 5 also show some discontinuities in the stress–strain curves. These discontinuities are more pronounced in the stress–strain curves between *σ*_*xx*_ and *σ*_*yy*_ versus *ɛ*_*xx*_ for constant *ɛ*_*yy*_ values, as shown in Figures 6A,B, where different color curves correspond to different *ɛ*_*yy*_ values, as shown with the corresponding colored legends in panel (B). These stress discontinuities are caused as an effect of the discontinuity of the first derivative of the energy with respect to *ɛ*_*xx*_, as Figure 6D shows for *ɛ*_*yy*_ = 0.13. In particular, for *ɛ*_*yy*_ = 0.13 (and strain values close to that), two minima of the energy curve as a function of strain appear, since *σ*_*xx*_ becomes zero twice (before and after the discontinuity), as Figure 6A shows. These minima can be also seen in Figure 6D.

The *ɛ*_*xx*_ values at which those discontinuities take place perfectly fit to a quadratic function of *ɛ*_*yy*_, as Supplementary Figure S1 shows the interatomic distance distributions for the discontinuity strain values reveals that those discontinuities take place when some second nearest neighbor distances (which for the CHON-2019 potential take the value of 2.459 Å at equilibrium), cross the value of 2.45 Å, as *ɛ*_*xx*_ decreases taking negative values for a constant *ɛ*_*yy*_ value. This is a strong indication that these discontinuities are probably due to some cutoff distance, which is used either by LAMMPS implementation of the ReaxFFs or the ReaxFFs themselves to define the bonds, by eliminating the bond order contributions for interatomic distances beyond that cutoff value. This indication is amplified by the fact that the interatomic distance *r*_*C*−*C*_ dependence of the carbon–carbon bond order, which is used in ReaxFFs, practically vanishes for *r*_*C*−*C*_ > 2.5 Å (see Ref. van Duin et al. (2001)). Moreover, for the saddle point of the ci-CH potential, which was discussed earlier in section 3.1.1, the distance between second nearest neighbors is 2.505 Å, which is very close to the 2.445 Å distance, which is found here. This indicates that maybe that saddle point and the second minimum, which appear in that case, are of the same origin as the discontinuity of the first derivative of the energy curve and the second energy minimum, respectively, which are shown in Figure 6D.

Interestingly, it appears that not only the stress, but also the energy curves have discontinuities, as Figure 6C shows, where the energy per atom versus *ɛ*_*xx*_ is plotted for constant *ɛ*_*yy*_ values. Those discontinuities appear in different strain *ɛ*_*xx*_ values compared with those already described and it is expected that they are also associated with similar cutoff distances and are also shown in Supplementary Figure S1. These energy discontinuities should possibly be considered in further improvements.

Similar discontinuities of the stress and the energy were also obtained using the C-2013 and GR-RDX-2021 potentials, as shown in the corresponding Supplementary Figure S1,S3.

These observations are in agreement with the findings of Furman and Wales (Furman and Wales (2019)), who also studied those discontinuities of ReaxFFs. According to their observations in the dissociation of N_2_, the bond energy term features a cusp at approximately 2.5Å, causing the first derivative of the bond energy to be discontinuous at this point. Those discontinuities were attributed to several bond order and bond distance cutoffs, in agreement with our suspicions. For the elimination of those discontinuities the authors proposed the use of tapering functions, which would allow a smooth transition between bonded and nonbonding environments. However, such tapering functions or any other way to eliminate those discontinuities are not yet included in the ReaxFFs.

To shed more light in those discontinuities, we examined which energy terms among those composing the CHON-2019 ReaxFF potential are responsible for those discontinuities. In the case of strained graphene six such terms exist, namely the atom, bond, valence angle, torsion angle, 4-body conjugation and van der Waals terms. We examined two cases, 1) one for which the energy is continuous and its first derivative *∂U*/*∂ɛ*_*xx*_ discontinuous, and 2) one for which the energy itself is discontinuous in a *ɛ*_*xx*_ range for constant *ɛ*_*yy*_. For the former we selected *ɛ*_*yy*_ = 0.13 and −0.054 ≤ *ɛ*_*xx*_ ≤ −0.052, corresponding to the energy curve shown in Figure 6D. For this case the discontinuity of *∂U*/*∂ɛ*_*xx*_ occurs at *ɛ*_*xx*_ = −0.0532. For the later we selected *ɛ*_*yy*_ = 0.18 and −0.094 ≤ *ɛ*_*xx*_ ≤ −0.092, where the energy is discontinuous more than once between *ɛ*_*xx*_ = −0.0929 and -0.0925. For the former the six energy terms are shown in the inset of Figure 6D, shifted by the value they have for *ɛ*_*xx*_ = −0.0540. Due to the small *ɛ*_*xx*_ range, all terms which are not responsible for the *∂U*/*∂ɛ*_*xx*_ discontinuity are expected to be linear functions of *ɛ*_*xx*_. Although the curves shown in the inset of Figure 6D give the impression of a linear dependence of those terms on *ɛ*_*xx*_, it is not linear for all terms. This can be seen in Figure 6E, which shows the residuals *δU*/*N* − *aɛ*_*xx*_ − *b* between the shifted energy values *δU*/*N* for each term and the fitting functions *aɛ*_*xx*_ + *b* of *δU*/*N* in the range before and after the discontinuity of *∂U*/*∂ɛ*_*xx*_, i.e. for −0.0540 ≤ *ɛ*_*xx*_ ≤ −0.0532 and for −0.0532 ≤ *ɛ*_*xx*_ ≤ −0.0520, respectively. If the dependence of *δU*/*N* on *ɛ*_*xx*_ is linear, then the two fitting functions should more or less coincide and the residuals, not only in the fitted interval, but also in the extrapolated interval should be practically zero. If, on the other hand, the residuals in the extrapolated interval is not zero, this means that the slope of the linear fitting function changes, indicating a discontinuous behavior of the derivative *∂U*/*∂ɛ*_*xx*_ of that energy term. As one can see in Figure 6E, the slope of the bond and atom energy terms change significantly, with a smaller slope change for the 4-body conjugation term. Consequently, the discontinuity of *∂U*/*∂ɛ*_*xx*_ is caused mainly due to the strain dependence of the bond and atom term of the energy, with a smaller contribution from the 4-body conjugation term. For the later the energy terms are depicted in Figure 6F, which shows that the discontinuity in that case clearly comes from the 4-body conjugation term. All other terms are continuous.

It is worth noting that the behavior described above is not associated to anomalous elongation of bonds, or rupture, which have been observed for large positive values of both *ɛ*_*xx*_ and *ɛ*_*yy*_. For graphene under strain along x and/or *y* direction there are only two kind of bonds with different lengths, namely those aligned along *x* direction, with bond length *a*, and those belonging to the zig-zag chains along the *y* direction, with bond length *b*. Moreover, there are two different types of bond angles, namely the angles *ϕ* formed between the bonds of those zig-zag chains, and the angles *θ* formed by the bonds of those zig-zag chains and the bonds directed along the *x* direction. The angles *ϕ* and *θ* depend with each other through the relation *θ* = *π* − *ϕ*/2. The discontinuities of the derivative *∂U*/*∂ɛ*_*xx*_ (i.e., the sharp change of the slope of the energy, like the one shown in Figure 6D) do not seem to affect the smooth change of *a*, *b* and *ϕ* as a function of strain *ɛ*_*xx*_. However, the discontinuities of *U*(*ɛ*_*xx*_) affects *a*, *b* and *ϕ*, which are also discontinuous as a function of *ɛ*_*yy*_. As examples of those cases we show in Supplementary Figure S4 the *a*, *b* and *ϕ* values of the equilibrium structure of graphene under strain *ɛ*_*xx*_ = 0.13 (left panels) and 0.18 (right panels) as a function of *ɛ*_*yy*_ in a *ɛ*_*yy*_ range, where for the former *U* is continuous and *∂U*/*∂ɛ*_*yy*_ discontinuous, and for the latter *U* is discontinuous.

#### 3.1.4 Phonon dispersion relation

**FIGURE 7:**
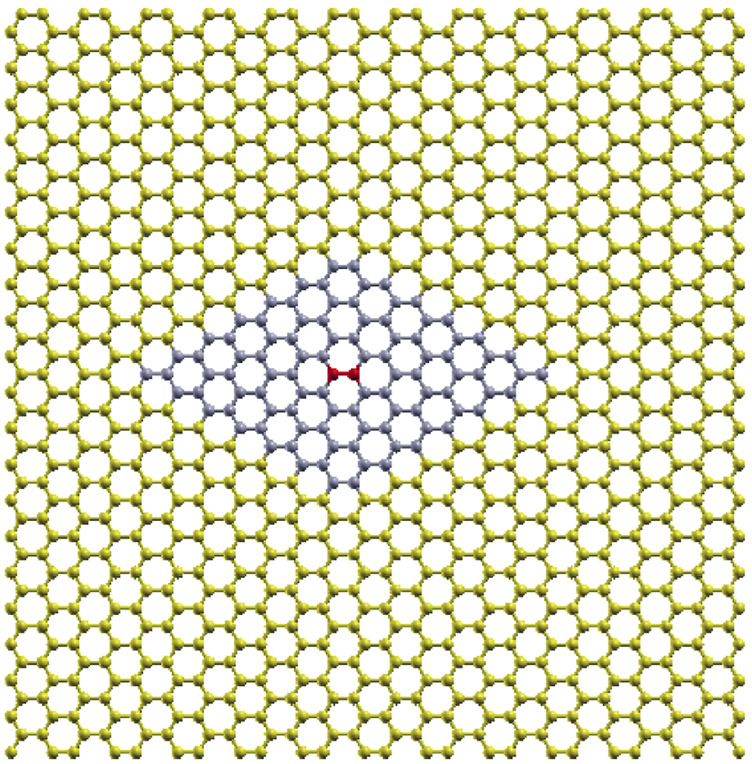
The 98 atom 7 × 7 hexagonal supercell (blue colored atoms) as part of the 1,008 atom rectangular supercell of Figure 1. The red colored atoms in the center of the 7 × 7 hexagonal supercell are those corresponding to the two-atom primitive unit cell of graphene, which are displaced from equilibrium for the necessary force calculations.

For the phonon dispersion relation we use an in-house code made by one of us, implementing the frozen phonons method^2^: The forces on all atoms of a 7 × 7 portion of the supercell, shown in Figure 7 (blue and red colored), are calculated for ± *δx*, ± *δy* and ± *δz* displacements of the atoms of the central unit cell (red colored). For our calculations, we chose *δx* = *δy* = *δz* = 0.0001 Å. Those forces are used to calculate numerically the second derivatives of the energy. E.g., for a displacement ± *δy*, one has

$$\frac{\partial^{2}U}{\partial x_{i}\partial y_{j}}\approx \frac{f_{i,x}\left(y_{j}-\delta y\right)-f_{i,x}\left(y_{j}+\delta y\right)}{2\delta y},$$ (11)

where *f*_*i*,*x*_(*y*_*j*_ ± *δy*) denotes the *x* component of the force **f**_*i*_ on *i*th atom. The *k* dependent dynamical matrix is then evaluated by discrete Fourier transform and diagonalized to get the different branches of dispersion relations.

The optimized 1008-atom rectangular supercell was used for the calculations. A smaller than a 7 × 7 force summation truncation introduces errors due to missing terms in the dynamic matrix, thus producing imaginary frequencies near the Γ point.

**FIGURE 8:**
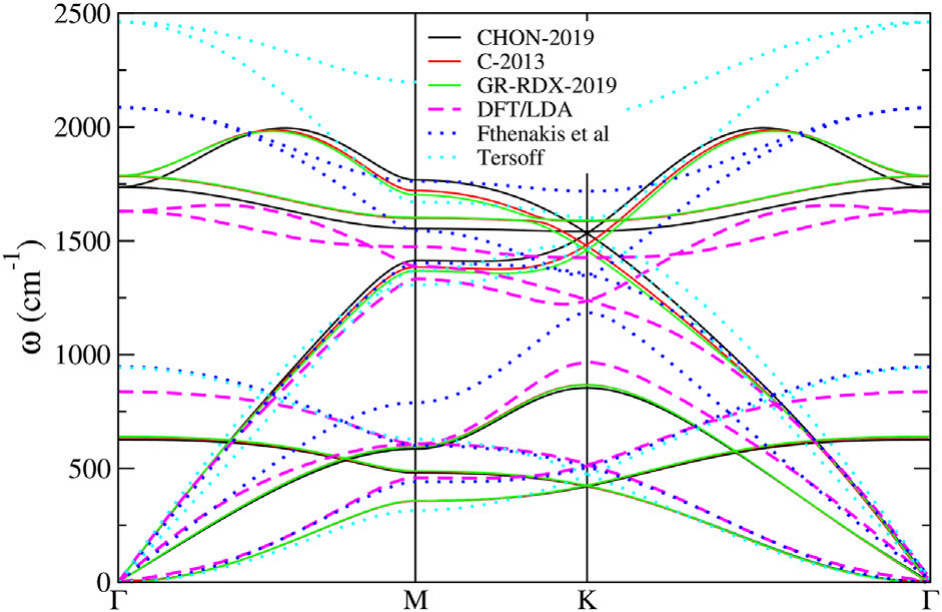
Phonon dispersion relation for the three ReaxFF potentials along ΓMKΓ path.

The phonon dispersion relations calculated for the potentials of the second group along the ΓMKΓ path in *k*-space are shown in Figure 8 and compared with 1) DFT results (Fthenakis et al. (2017)), 2) the results of the much simpler molecular mechanics potential of Fthenakis *et al* (Fthenakis et al. (2017)) and 3) the Tersoff potential (Tersoff (1988)). As one can see in that figure, the phonon dispersion relations provided by the three potentials are very similar. Quantitative agreement with DFT dispersion relations can also been observed at several parts of the ΓMKΓ path, indicating that these potentials are an improvement with respect to Tersoff and Fthenakis, especially for the representation of the in-plane optical branches. Conversely, the representation of the flexural modes at the zone boundary and of the optical out of plane modes is worse than in Tersoff and Fthenakis.

### 3.2 Carbon nanotubes

#### 3.2.1 Energetics

It has been shown that the energy of single-wall carbon nanotubes (CNTs) depends on the diameter *D* of the CNT, as an effect of the CNT curvature as follows (Tibbetts (1984); Fthenakis et al. (2017); Kanamitsu and Saito (2002)).

$$U/N=U_{0}+C_{1}/D^{2}+C_{2}/D^{4}$$ (12)

where *U*_0_ is the energy of graphene corresponding to *D* = *∞*. The *C*_1_ term is dominant, but *C*_2_ is not negligible. Depending on the method used for the energy calculations, the value of *C*_1_ ranges between ≈5 and ≈10 eV⋅Å^2^ (see Ref. Fthenakis et al. (2017) and references therein).

**FIGURE 9:**
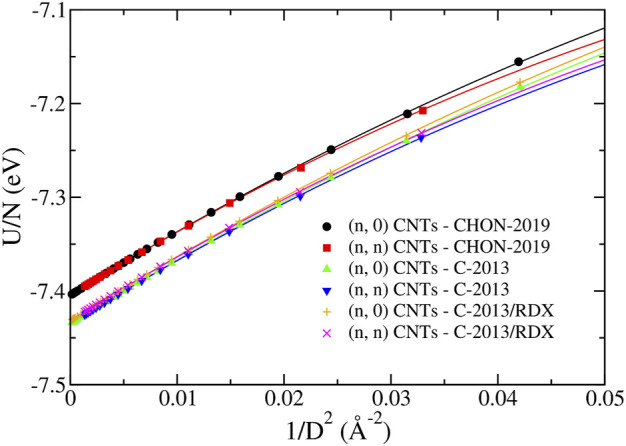
The energy per atom *U*/*N* of the (*n*, 0) and (*n*, *n*) CNTs predicted by the CHON-2019, C-2013 and GR-RDX-2021 potentials. The lines correspond to the fitting lines provided in Tab. 2.

In Figure 9, we report the energy of the (*n*, *n*) and the (*n*, 0) CNTs as a function of their diameter *D*, obtained with the three ReaxFF potentials of the second group. The fit with Eq. 12 is reported in Table 2 showing that *U*_0_ differs from the binding energy of graphene reported in Table 1 by less than 0.004%. The main differences between the curves in Figure 9 are due to this parameter, which differs by 0.03 eV in CHON-2019 potential with respect to others. The parameter *C*_1_ ≈ 7 eV⋅Å^2^ for all three potentials and for both (*n*, 0) and (*n*, *n*) CNTs. Moreover, *C*_2_ turns out to be different for (*n*, 0) and (*n*, *n*) CNT’s ( ≈−25 eV⋅Å^4^ and ≈−30 eV⋅Å^4^, respectively) without significant differences between the three potentials.

**TABLE 2:** Fitting functions of the energy per atom *U*/*N* of the (*n*, 0) and (*n*, *n*) CNTs for the three ReaxFF potentials. *U*/*N* and the CNT diameter *D* are given in eV and Å units, respectively.

CNTs	Potential	Equation *U*/*N* =
(*n*, 0)	CHON-2019	−7.4043 + 7.0162/*D* ^2^ − 26.339/*D* ^4^
(*n*, 0)	C-2013	−7.4346 + 7.0060/*D* ^2^ − 24.683/*D* ^4^
(*n*, 0)	GR-RDX-2021	−7.4315 + 7.0798/*D* ^2^ − 24.900/*D* ^4^
(*n*, *n*)	CHON-2019	−7.4043 + 7.0385/*D* ^2^ − 31.710/*D* ^4^
(*n*, *n*)	C-2013	−7.4345 + 6.9853/*D* ^2^ − 29.267/*D* ^4^
(*n*, *n*)	GR-RDX-2021	−7.4315 + 7.0732/*D* ^2^ − 30.212/*D* ^4^

#### 3.2.2 Mechanical properties and response to strain

**FIGURE 10:**
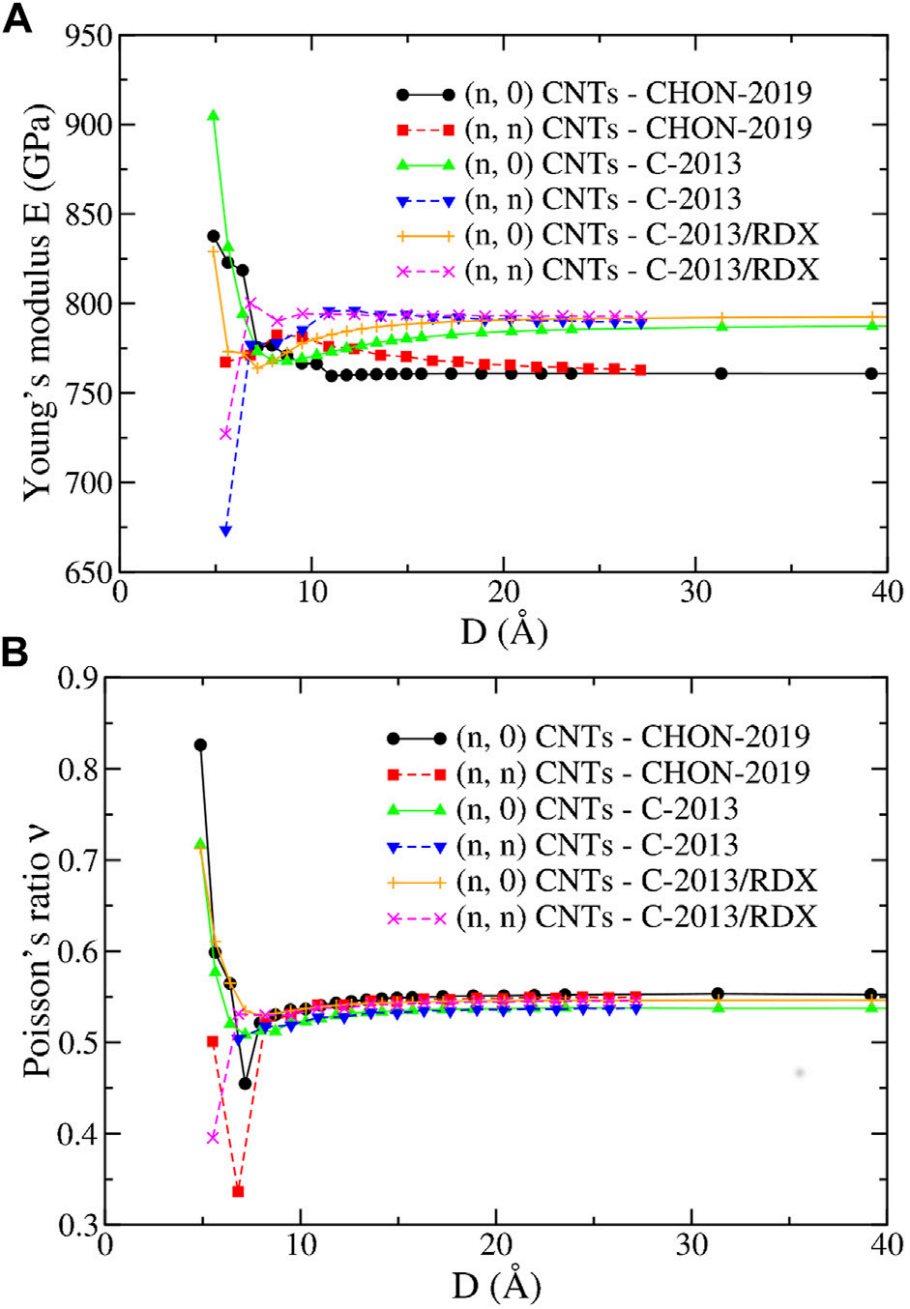
**(A)** The Young’s modulus *E* and **(B)** the Poisson’s ratio *ν* as a function of the diameter *D* for the (*n*, 0) and (*n*, *n*) CNTs of the present study predicted by the CHON-2019, C-2013 and GR-RDX-2021 potentials.

Figure 10 reports *E* and *ν* of the (*n*, 0) and (*n*, *n*) CNTs obtained with the ReaxFF potentials of the second group. For *D* > 10 Å, *E* and *ν* have a monotonic behavior ultimately leading to the “bulk” graphene values, and independent from the chirality, as expected, and also seen in DFT calculations (Ogata and Shibutani (2003); Qian et al. (2021)). Consequently, as in the “bulk”, *E* and *ν* are underestimated and overestimated, respectively, compared DFT calculations. On the other hand, they are coherent with the values obtained with molecular dynamics using C-2013 by Jensen *et al* (Jensen et al. (2015)) for the (20.0) (*E* = 777 GPa) and by Qian *et al* (Qian et al. (2021)) for the (5.5) (*E* = 764 GPa) and for the (10.0) (*E* = 825 GPa).

Conversely, the trend for *D*⪅10 Å is less clear displaying increasing or decreasing behavior and especially opposite divergences at small *D* depending on the potential, as previously observed (see Ref. Sakharova et al. (2017) and references therein). On the other hand, *ν* displays a regularly increasing behavior for all three potentials and for both (*n*, 0) and (*n*, *n*) CNTs, for *D*⪆10 Å, and a minimum at small *D* whose location and depth is dependent on the potential. However, both the dependence of *E* and *ν* on *D* > 10 Å is rather weak, in accordance with similar studies using other potentials (Fthenakis et al. (2017)).

**FIGURE 11:**
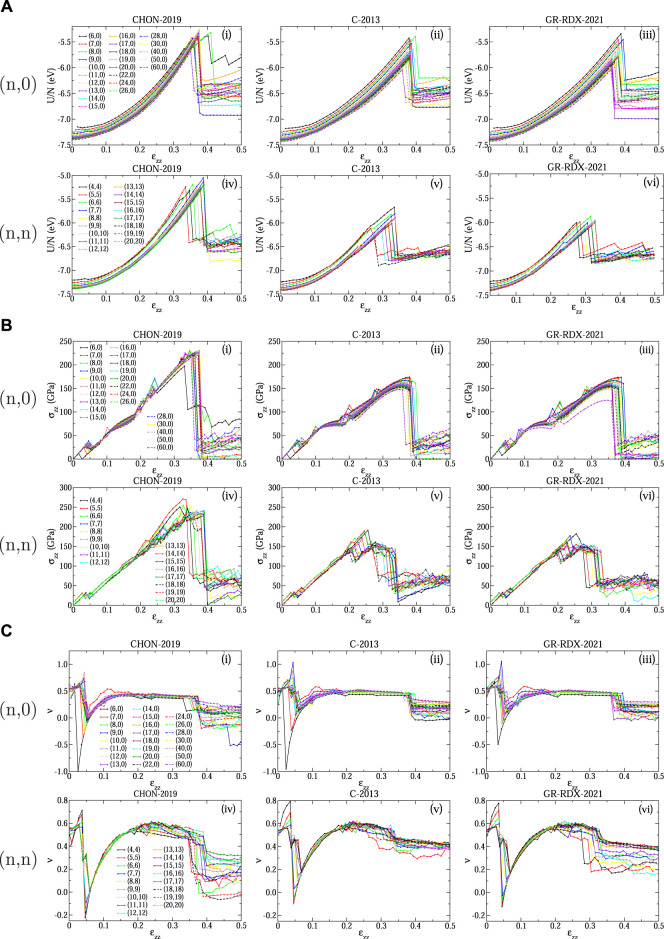
Response of CNTs to strain: **(A)** Energy per atom *U*/*N*, **(B)** stress *σ*_*zz*_ and **(C)** Poisson’s ratio *ν* as a function of strain *ɛ*_*zz*_ for (*n*, 0) (subpanels i, ii, iii) and (*n*, *n*) (subpanels iv, v, vi) CNTs predicted using the CHON-2019 (subpanels i, iv), C-2013 (subpanels ii, v) and GR-RDX-2021 (subpanels iii, vi) potential.

Panels (A), (B) and (C) of Figure 11 show the energy per atom *U*/*N*, the stress *σ*_*zz*_ and the Poisson’s ratio *ν*, respectively, of (*n*, 0) and (*n*, *n*) CNTs, calculated using the three ReaxFF potentials, as a function of the strain *ɛ*_*zz*_ in the range [0, 0.5]. The “drops” shown in Figure 11B at *ɛ*_*zz*_ ≈ 0.05 are likely to be of the same origin as the corresponding ones discussed for graphene. Accordingly, the plots of *ν* shown in Figure 11C are very similar with their counterpart for graphene (Figure 4B). There are some differences due to the CNT curvature, mostly affecting CNTs with small diameter. These “drops” for the C-2013 potential can be also seen in the stress - strain plots of the study by Qian *et al* (Qian et al. (2021)) for the (5.5) and the (10.0) CNTs.

#### 3.2.3 Ultimate tensile strength and fracture strain limits

**FIGURE 12:**
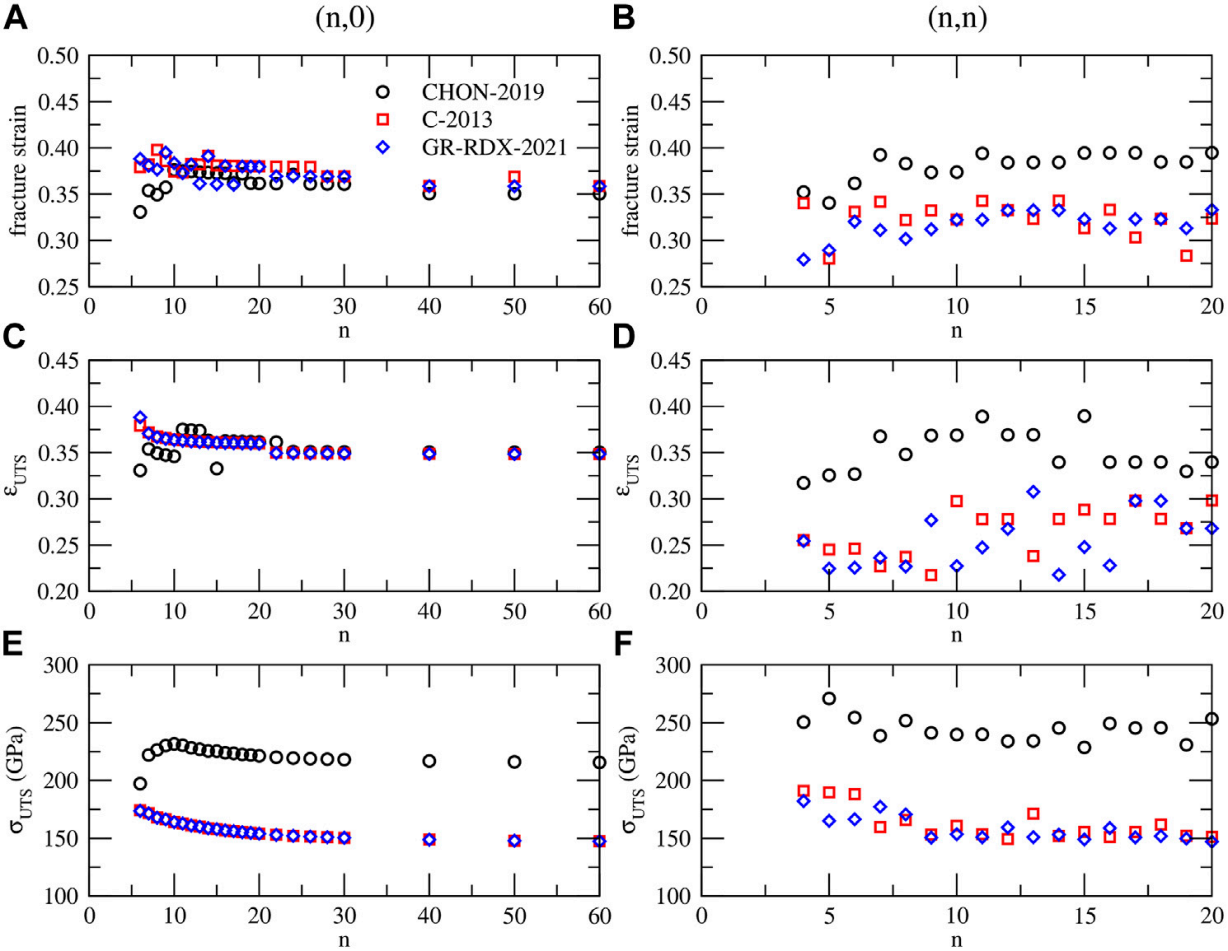
**(A)** and **(B)** fracture strain, **(C)** and **(D)** ultimate tensile strain *ɛ*_*UTS*_, **(E)** and **(F)** ultimate tensile strength *σ*_*UTS*_ for (*n*, 0) (left panels - **(A)**, **(C)** and **(E)**) and (*n*, *n*) (right panels - **(B)**, **(D)** and **(F)**) CNTs, predicted by CHON-2019 (black circles), C-2013 (red squares) and GR-RDX-2021 (blue diamonds).

The energy and stress drops shown in Figures 11A,B at high strain values indicate brittle fractures. The ultimate tensile strength *σ*_*UTS*_ (i.e., the maximum stress that can be reached as strain increases) and the corresponding strain *ɛ*_*UTS*_, as well as the fracture strain limits *ɛ*_*F*_, defined as the strain at which the energy drops occur, are shown in Figure 12 versus *n* of the (*n*, 0) and (*n*, *n*) CNTs.

As shown in Figures 11A,B the two values *ɛ*_*UTS*_ and *ɛ*_*F*_, coincide in many cases, or *ɛ*_*F*_ is slightly larger. For the (*n*, 0) CNTs they both range between 0.33 and 0.40, with most of the cases ranging between 0.36 and 0.38 for *ɛ*_*F*_, and 0.35 and 0.37 for *ɛ*_*UTS*_, with not significant differences between the three potentials. For the (*n*, *n*) CNTs the *ɛ*_*F*_ and *ɛ*_*UTS*_ values are more scattered. Those obtained with CHON-2019 fall in a similar strain range as those for the (*n*, 0) CNTs, i.e. 0.36–0.39 for *ɛ*_*F*_ and 0.32–0.39 for *ɛ*_*UTS*_, while those from C-2013 and GR-RDX-2021 are smaller for *ɛ*_*F*_ (0.28–0.34) and much smaller for *ɛ*_*UTS*_ (0.22–0.31). Concerning *σ*_*UTS*_, values predicted by CHON-2019 both for (*n*, 0) and (*n*, *n*) CNTs are higher than those predicted by C-2013 and GR-RDX-2021, and sligthly decreasing with *n*, although the values for (*n*, *n*) CNTs (panel f), are more scattered. Moreover, *σ*_*UTS*_ from CHON-2019 for (*n*, 0) CNTs ( ≈230 GPa) are smaller than those for the (*n*, *n*) ( ≈250 GPa), while those from C-2013 and GR-RDX-2021 appear almost independent from chirality (*σ*_*UTS*_ ≈ 160 GPa). These values of strain fall into the experimental range, which, on average is not larger than ≈20 %, and can be as small as 2%, while *σ*_*UTS*_ does not exceed the value of ≈150 GPa (Walters et al. (1999); Yu et al. (2000); Demczyk et al. (2002)), although in some specific experimental conditions much larger values (Bozovic et al. (2003); Troiani et al. (2003)) up to 280% (Huang et al. (2006)) have been reported for *ɛ*.

On the theoretical side, Ogata and Shibutani (Ogata and Shibutani (2003)) reported a study on (8,0), (9,0), (10,0) and (8,8) CNTs finding *σ*_*UTS*_ ∼ 86.8–95.6 GPa (with tight binding, (TB)) and 117.4–114.6 GPa (with DFT), and *ɛ*_*UTS*_ ∼0.170–0.211 (with TB) and 0.108–0.110 (with DFT). DFT calculations by Qian *et al* (Qian et al. (2021)) provide similar results, ≈100 GPa for the (10.0), ≈92 GPa for the (5.5) CNT, and *ɛ*_*UTS*_ = 0.22 for the (5.5) CNT, while for the (10.0) CNT the *ɛ*_*UTS*_ was found significantly larger (*ɛ*_*UTS*_ = 0.33). Indeed, the values obtained with classical potentials, are found, on average, larger than DFT values. Duan *et al* (Duan et al. (2007)) reported *ɛ*_*UTS*_ = 0.2736–0.4349 and *σ*_*UTS*_ = 99.89–134.01 GPa for (10,*n*) CNTs, *n* = 0, 1, 3, 5, 7, 9, 10, using four classical potentials (COMPASS, a modified Morse, REBO and Dumitrica potentials) while Qian *et al* (Qian et al. (2021)) found *σ*_*UTS*_ = 90–407.1 GPa, and *ɛ*_*UTS*_ 0.19–0.56 for (10.0) and (5.5) CNTs, using eight classical potentials (Tersoff and modified Tersoff, AIREBO and modified AIREBO, EDIP, LCBOP, C-2013 and GAP-20). Therefore the results provided by the three ReaxFF potentials of the present study are in line with those of other classical potentials, performing even slightly better in comparison to DFT. On the other hand, the comparison to experiment is very difficult given the large spread of values.

### 3.3 Fullerenes and the pentagon adjacency penalty rule

In this part, we calculate the energy *U* of the icosahedral C_60_ fullerene and the energy of the 40 isomers of C_40_ fullerene, which can be obtained with all possible arrangements of pentagonal and hexagonal rings of the C_40_ fullerene structure (see Refs. Fowler and Manolopoulos (1995), Albertazzi et al. (1999) and Fthenakis et al. (2017)). Albertazzi *et al* (Albertazzi et al. (1999)) showed that a simple descriptor of the energy of those isomers is the number *N*_*p*_ of pentagon adjacencies and that the energy *U* of those isomers can be approximated by

$$U=aN_{p}+b$$ (13)

the parameter *a* ranging between 20 and 100 kJ/mol for different calculations, while for the C_40_ isomers *N*_*p*_ ranges between 10 and 20. Therefore, the maximum energy difference Δ*U* = *a*Δ*N*_*p*_ of those isomers can be estimated for the maximum values Δ*N*_*p*_ = 10 and *a* = 100 kJ/mol and is of the order of 1,000 kJ/mol (10 eV or 0.25 eV/atom). Similarly, the energy difference between isomers for which the *N*_*p*_ value differs by one can be estimated as 0.025 eV/atom, or even smaller down to 5 meV/atom, which gives an idea of the required sensitivity of the used force field.

At variance with graphene, fullerenes have non-zero torsional angles. In a recent work (Fthenakis et al. (2017)), Fthenakis *et al* showed the importance of torsional terms in the energy expression of the molecular mechanics potential for sp^2^ carbon systems. Neglecting those terms turns out in a poor dependence of the energy of the C_40_ fullerene isomers on *N*_*p*_, and a strong underestimation of the isomers’ energy differences. This may explain the small value of *a* found by Albertazzi *et al* with the Tersoff (*a* = 24.4 kJ/mol) and the Brenner (*a* = 36.1 kJ/mol) potentials, which do not explicitly include torsional terms. On the other hand, potentials which include torsional terms, like those by Fthenakis *et al* (Fthenakis et al. (2017)) or DTMM (Crabbe et al. (1994)) used by Albertazzi *et al* (Albertazzi et al. (1999)), return higher slope values, although not as high as those given by *ab initio* and semi-empirical methods, which are of the order of 80–100 kJ/mol (see Ref. Albertazzi et al. (1999)).

**FIGURE 13:**
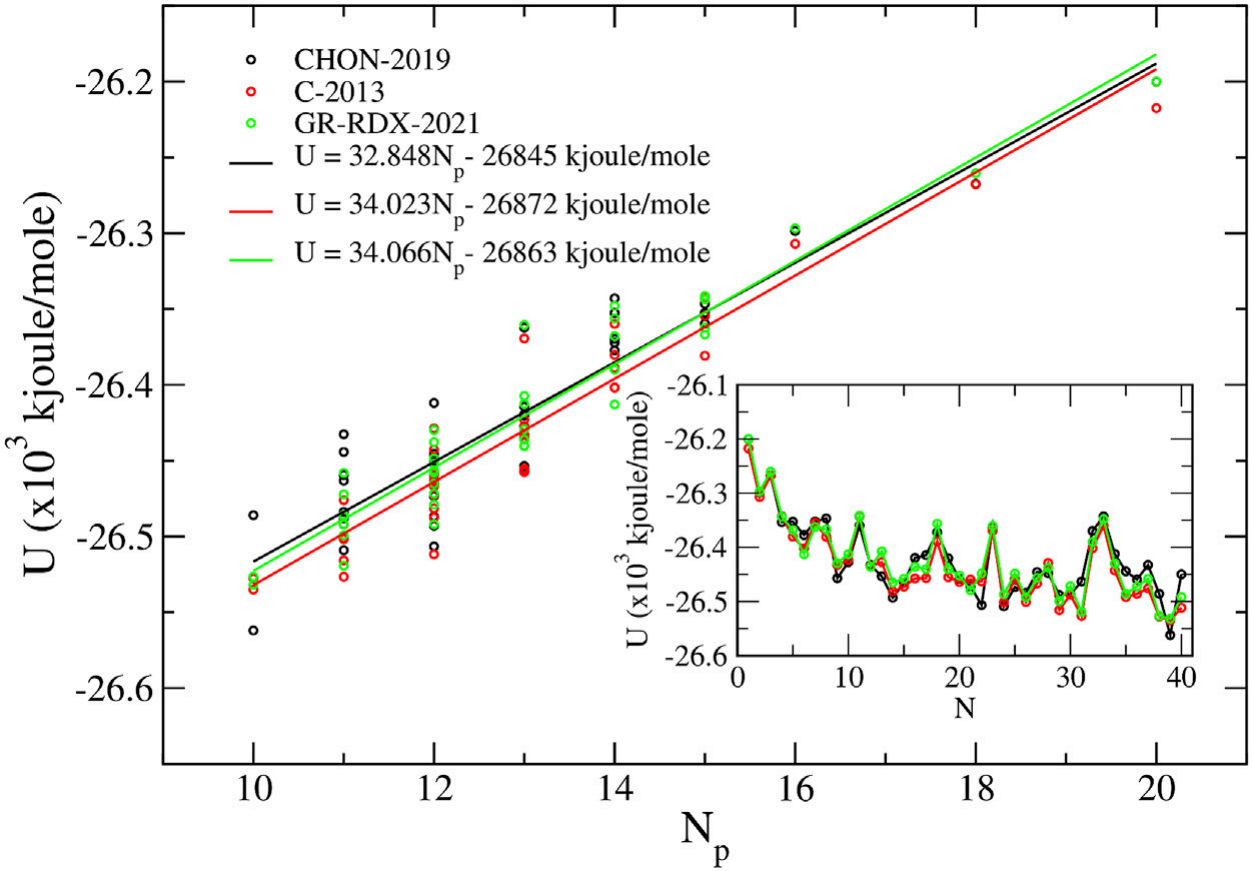
Binding energy *U* of the 40 C_40_ fullerene isomers vs*.* the pentagon adjacencies *N*_*p*_ calculated using the three ReaxFF potentials. In the inset, we show the calculated energy values versus the standard C_40_ isomer enumeration (Fowler and Manolopoulos (1995).

In Figure 13, we report the energy of the C_40_ isomers as a function of *N*_*p*_ for the three ReaxFF potentials of the second group. The results are compatible with a linear behavior with *a* = 32.848, 34.023 and 34.066 kJ/mol for CHON-2019, C-2013 and GR-RDX-2021, respectively, with correlation coefficient *R* ^2^ values of 0.860, 0.918 and 0.922, respectively. These values of *a* are comparable with those found by the molecular mechanics potentials of Fthenakis *et al* (Fthenakis et al. (2017)) (*a* = 40.5 kJ/mol) and the DTMM potential (Crabbe et al. (1994)) (*a* = 42.2 kJ/mol), i.e., they are smaller than DFT values, which seems to be a problem, common to all classical potentials, as confirmed by a study (Aghajamali and Karton (2021)) comparing the energy of the 1812 C_60_ isomers by DFT to those obtained using several potentials including the CHO and the C-2013.

The inset of Figure 13 shows *U* as a function of standard isomers enumeration, *N*, used also in Refs. (Fowler and Manolopoulos (1995)), (Albertazzi et al. (1999)) and (Fthenakis et al. (2017)), clearly showing a high correlation among energies predicted by the three ReaxFF potentials of the second group. The optimized C_40_ isomers using the GR-RDX-2021 potential are shown in Supplementary Figure S5. The isomer number (40:*N*) is shown below each structure. It is worth noting that energy differences among the three potentials are less than in graphene. The maximum energy difference per atom in any isomer evaluated with the three potential is of the order of at most 0.016 eV, and 0.003 eV on the average. The corresponding difference for graphene is 0.03 eV/atom (see above).

Among the 12 potentials used in the study by Albertazzi *et al* (Albertazzi et al. (1999)), all predict isomer no 38 to be the most stable, except Tersoff. Unfortunately, even the three ReaxFF potentials of the second group fail to predict isomer 38 as the most stable. CHON-2019 potential predicts isomer no 39 to be the most stable one, with no 30, 29, 24, 22 and 14 having energies between isomers no 39 and no 38. The energy difference between isomers no 39 and 38 is 76 kJ/mol, corresponding to 0.78 eV (or 20 meV/atom). C-2013 and GR-RDX-2021 potentials work better in this respect, predicting no 39 to be the most stable one, followed by isomer no 38 with a very small energy difference, 2.0 meV/atom for C-2013 and 1.4 meV/atom for GR-RDX-2021. For completeness, we report that Fthenakis *et al* (Fthenakis et al. (2017)) find isomer no 38 as the most stable one.

As a final validation check, we evaluated the energy of the icosahedral C_60_ and its difference with respect to graphene using the three potentials of the second group. The values we find with CHON-2019, C-2013 and GR-RDX-2021 potentials, are 0.3625, 0.3759 and 0.3767 eV/atom, respectively, while the corresponding experimental value (Fthenakis (2013); Chen et al. (1991)) is 0.41 ± 0.02 and the one calculated with DFT at the GGA/PBE level (Wirz et al. (2016)) is 0.38 eV/atom. Therefore, the three ReaxFF potentials, although in some cases they fail to capture the very small energy differences between the C_40_ fullerene isomers, they qualitative reproduce the linear increase of the fullerene energy with *N*_*p*_ and correctly predict the relative energy of the icosahedral C_60_ fullerene with respect to graphene.

## 4 Conclusion

In this work, we study the performance of 11 ReaxFF potentials in reproducing the structural, vibrational and mechanical properties of fullerene, graphene and carbon nanotubes under strain up to the rupture limit. Ten of them are commonly used general reactive potentials. The 11th one, which we call GR-RDX-2021, is built in this work combining the C-2013 and RDX. The combination is realized defining two different types for carbon atoms belonging to graphene (C_*g*_) or other molecules (C) which are treated with C-2013 and RDX, respectively, to exploit their capabilities to treat graphene and other molecules, respectively, so to optimize the overall performance of the representation. This procedure does not require any further reparameterization and can be easily generalized to any couple of potentials at least in the same class.

According to our findings, seven of these potentials predict unphysical values of the Poisson’s ratio describing practically unstretchable graphene bonds in comparison with bond angle deformation. These potentials are not indicated for studies of the elastic properties of sp^2^ systems. We the focus our study on they four remaining potentials, C-2013, CHO-2016, CHON-2019 and the new one GR-RDX-2021 which do not suffer from such problems. We evaluated the structural and mechanical properties of graphene, its response to tensile stain and its phonon band structure. We studied the energetic and mechanical properties of the (*n*, 0) and (*n*, *n*) CNTs as a function of their diameter, as well as, their response on tensile strain and fracture. We studied the energetics of the 40 C_40_ fullerene isomers and the predictions of the pentagon adjacency penalty rule, as well as, the energy of the icosahedral C_60_ fullerene. The hybrid GR-RDX-2021 potential is tested here on its performances on stretched graphene-derived materials, and is intended to be used to explore the consequences of stretching on the reactivity of graphene with organic molecules including H, O and N, besides C, where it is expected to provide a description at least as good as its parent potential RDX. These aspects are the matter of a fortcoming paper.

Overall, the selected ReaxFF potentials predict practically the same values for graphene bond length (1.42 Å) and cohesive energy (-7.4 eV/atom), in agreement with experimental and DFT values. They also predict similar phonon dispersion relations for graphene, whose discrepancy from DFT is on average similar. Moreover, they all qualitatively and correctly predict: 1) The trend for the energy of CNTs as a function of diameter, 2) the increasing trend of the energy of fullerene isomers as a function of their pentagon adjacencies, and 3) the correct energy difference between the icosahedral C_60_ fullerene with respect to graphene. On the other hand, they underestimate the Young’s modulus of both graphene and CNTs by ≈3/4 and overestimate their Poisson’s ratio by ≈3 times, compared to DFT. In terms of the bond stretching and bond angle bending “spring constants” of an equivalent sticks-spiral mechanical model, the bond stretching is stronger than that provided by DFT by a factor of ≈4/3 , while the bond angle bending is weaker by a factor of ≈1/2 .

We found unexpected drops and discontinuities in the stress–strain plots, both for graphene and CNT’s. We then performed an accurate analysis of the PES as a function of *ɛ*_*xx*_ and *ɛ*_*yy*_. Our study revealed several irregularities of the PES (*U*), including 1) discontinuities of stresses due either to discontinuities of *U* or its gradients 2) the existence of more than one minimum of *U* at constant *ɛ*_*xx*_ or *ɛ*_*yy*_ and 3) changes of the “spring constant” of the equivalent stick-spiral model. Three different regions of the *U* landscape and corresponding regimes are identified, whose borders crossing is related to the “drops” in the stress–strain plots. Due to those drops, which significantly affect the structural behavior of graphene and CNTs under strain, the dependence of *ν* on *ɛ*, for *ɛ* > 0.03 is rather irregular.

The predictions of the three ReaxFF potentials for the fracture strain, the ultimate tensile strength and the corresponding strain values for the CNTs are overestimated with respect to DFT results. They are, however, in the range of the predictions of other potentials and even closer to the DFT, and fall within the experimental range, which displays a very large variability.

The overall conclusion, therefore, is that the studied ReaxFF potentials, for strain values ⪅0.05, provide quantitatively reliable results, for the energy and structural properties of graphene, the energetics of CNTs and fullerenes, as well as, the phonon band structure of graphene. Those results are comparable with the corresponding ones of other non-reactive potentials and DFT calculations. On the other hand, further studies are needed at larger stress values. On a qualitative level, reasonable behavior is reproduced displaying the onset of plasticity and rupture, while the quantitative aspects needs further investigations.

The present study, therefore, provides interesting information for the strengths and weakness of those potentials, which hopefully will be useful for their further improvement.

## Data Availability

The original contributions presented in the study are included in the article/Supplementary Materials, further inquiries can be directed to the corresponding author.

## Footnotes

1 For routine runs workstations. Thousands on High Performance Computing systems.

2 The code is available upon request.
