# Supplementary material for: Evaluating the performance of ReaxFF potentials for sp2 carbon systems (graphene, carbon nanotubes, fullerenes) and a new ReaxFF potential
Source: Front Chem. 2022 Aug 29;10:951261. doi: 10.3389/fchem.2022.951261 (PMC9465816; doi:10.3389/fchem.2022.951261)
Supplement: Supplementary file 1 [file DataSheet2.PDF]

Supplementary Material for "Evaluating the performance of ReaxFF potentials for sp^2 carbon systems (graphene, carbon nanotubes, fullerenes) and a new ReaxFF potential"

Zacharias G. Fthenakis [1,2,3,4,5], Ioannis D. Petsalakis [2], Valentina Tozzini [1,5] and Nektarios N. Lathiotakis [2]

[1]Istituto Nanoscienze-CNR, Pisa, Italy
[2]Theoretical and Physical Chemistry Institute, National Hellenic Research Foundation, Athens, Greece
[3]Department of Surveying and Geoinformatics Engineering, University of West Attica, Athens, Greece
[4]Department of Marine Engineering, University of West Attica, Athens, Greece
[5]NEST, Scuola Normale Superiore, Pisa, Italy

ABSTRACT OF THE MAIN MANUSCRIPT

We study the performance of eleven reactive force fields (ReaxFF), which can be used to study sp^2 carbon systems. Among them a new hybrid ReaxFF is proposed combining two others and introducing two different types of C atoms. The advantages of that potential are discussed. We analyze the behavior of ReaxFFs with respect to (i) the structural and mechanical properties of graphene, its response to strain and phonon dispersion relation; (ii) the energetics of $(n, 0)$ and (n, n) carbon nanotubes (CNTs), their mechanical properties and response to strain up to fracture; (iii) the energetics of the icosahedral C_{60} fullerene and the 40 C_{40} fullerene isomers. Seven of them provide not very realistic predictions for graphene, which made us focusing on the remaining, which provide reasonable results for (i) the structure, energy and phonon band structure of graphene, (ii) the energetics of CNTs versus their diameter and (iii) the energy of C_{60} and the trend of the energy of the C_{40} fullerene isomers versus their pentagon adjacencies, in accordance with density functional theory (DFT) calculations and/or experimental data. Moreover, the predicted fracture strain, ultimate tensile strength and strain values of CNTs are inside the range of experimental values, although overestimated with respect to DFT. However, they underestimate the Young's modulus, overestimate the Poisson's ratio of both graphene and CNTs and they display anomalous behavior of the stress - strain and Poisson's ratio - strain curves, whose origin needs further investigation.

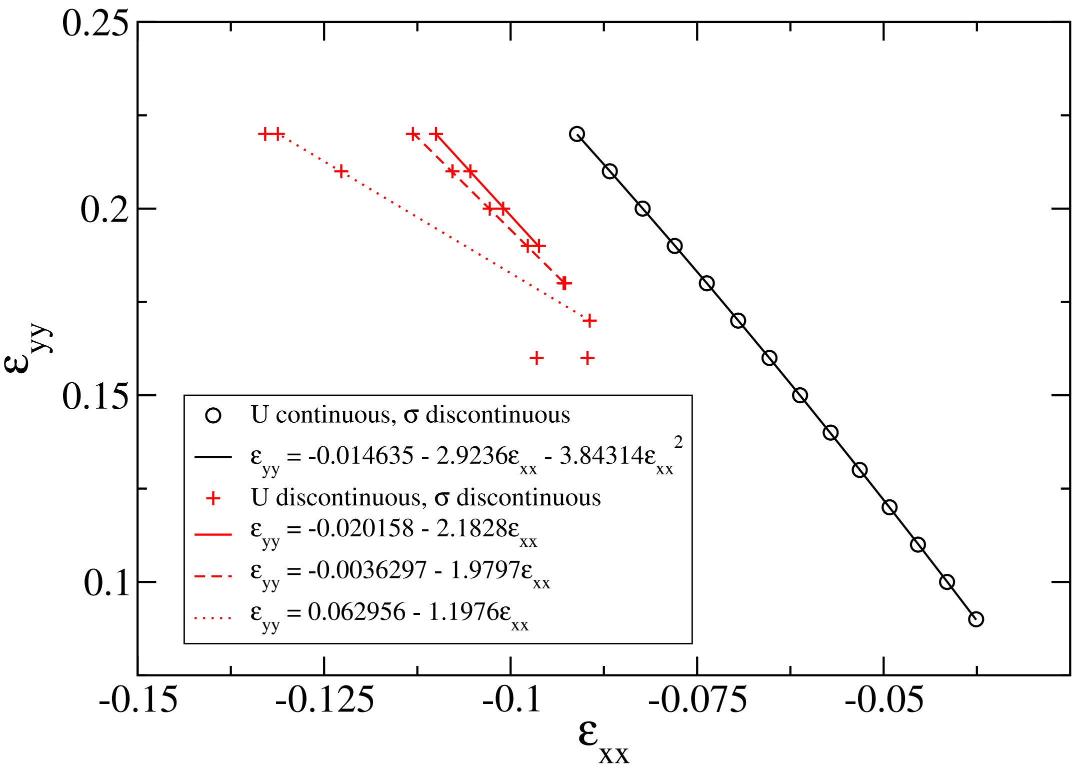

Figure S1. Strain ε_{xx} and ε_{yy} values at which discontinuities occur for the CHON-2019 ReaxFF potential. The (ε_{xx}, ε_{yy}) points for which the energy is continuous and the stress discontinuous (black circles) perfectly fit to a quadratic curve. The (ε_{xx}, ε_{yy}) points for which both the energy and the stress are discontinuous (red crosses) perfectly fit to straight lines.

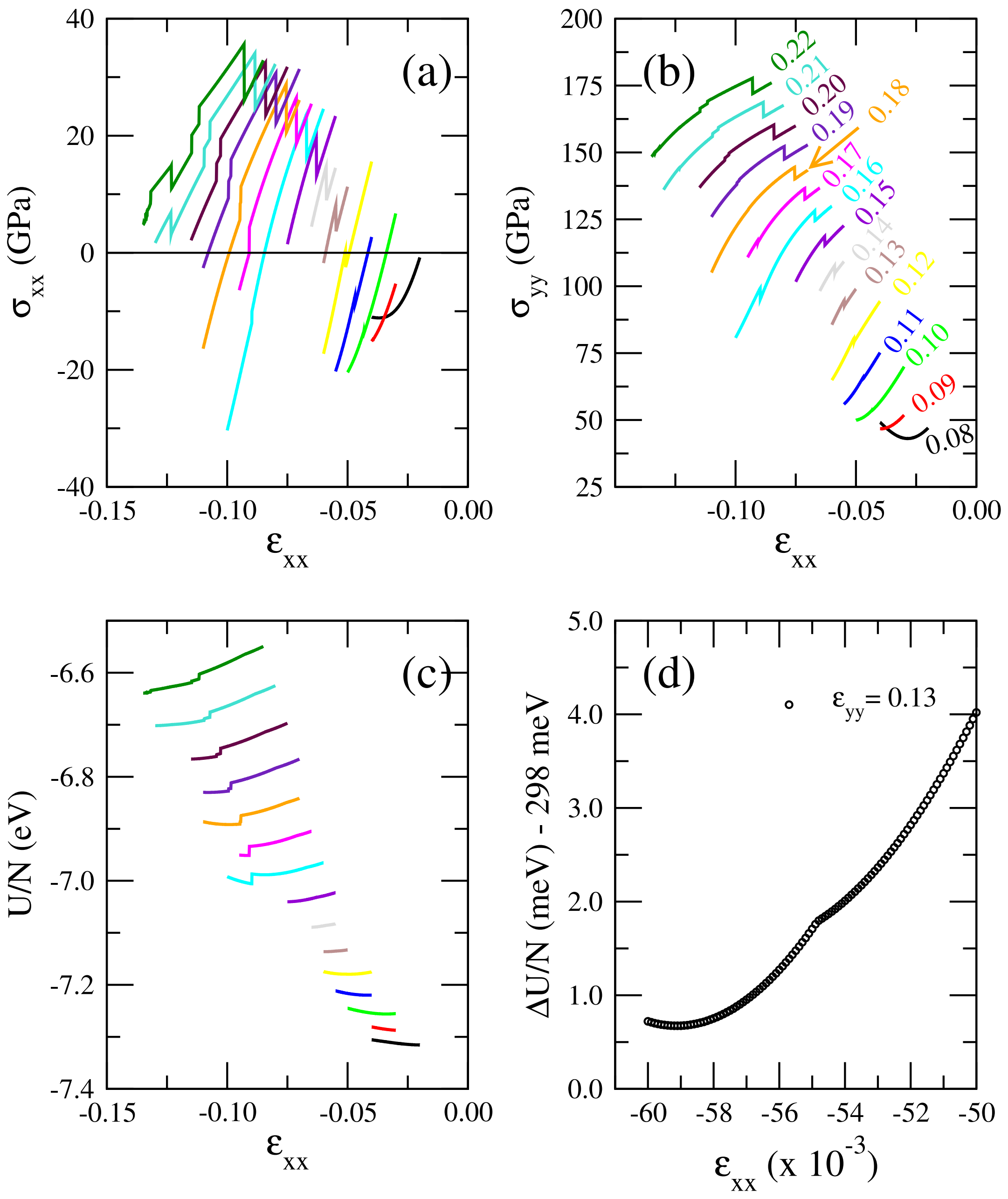

Figure S2. "Jumps" (discontinuities) in stress and energy plots obtained using the C-2013 potential. (a) Stress along x direction (σ_{xx}), (b) stress along y direction (σ_{yy}) and (c) energy per atom (U/N) as a function of strain along x direction (ε_{xx}) for fixed ε_{yy} values indicated with different colors. The ε_{yy} values corresponding to each curve are shown next to each curves in panel (b) with the same color. (d) Strain energy per atom $\Delta U/N = U/N - U_{coh}$ shifted by 298 meV versus ε_{xx} for $\varepsilon_{yy} = 0.13$ showing the discontinuous change in its slope. Contrary to CHON-2019 potential, there the two energy minima do not appear for $\varepsilon_{yy} = 0.13$. They appear, however, for $\varepsilon_{yy} = 0.12$, as panel (a) shows. The ε_{xx} increment in these plots is 10^{-4}.

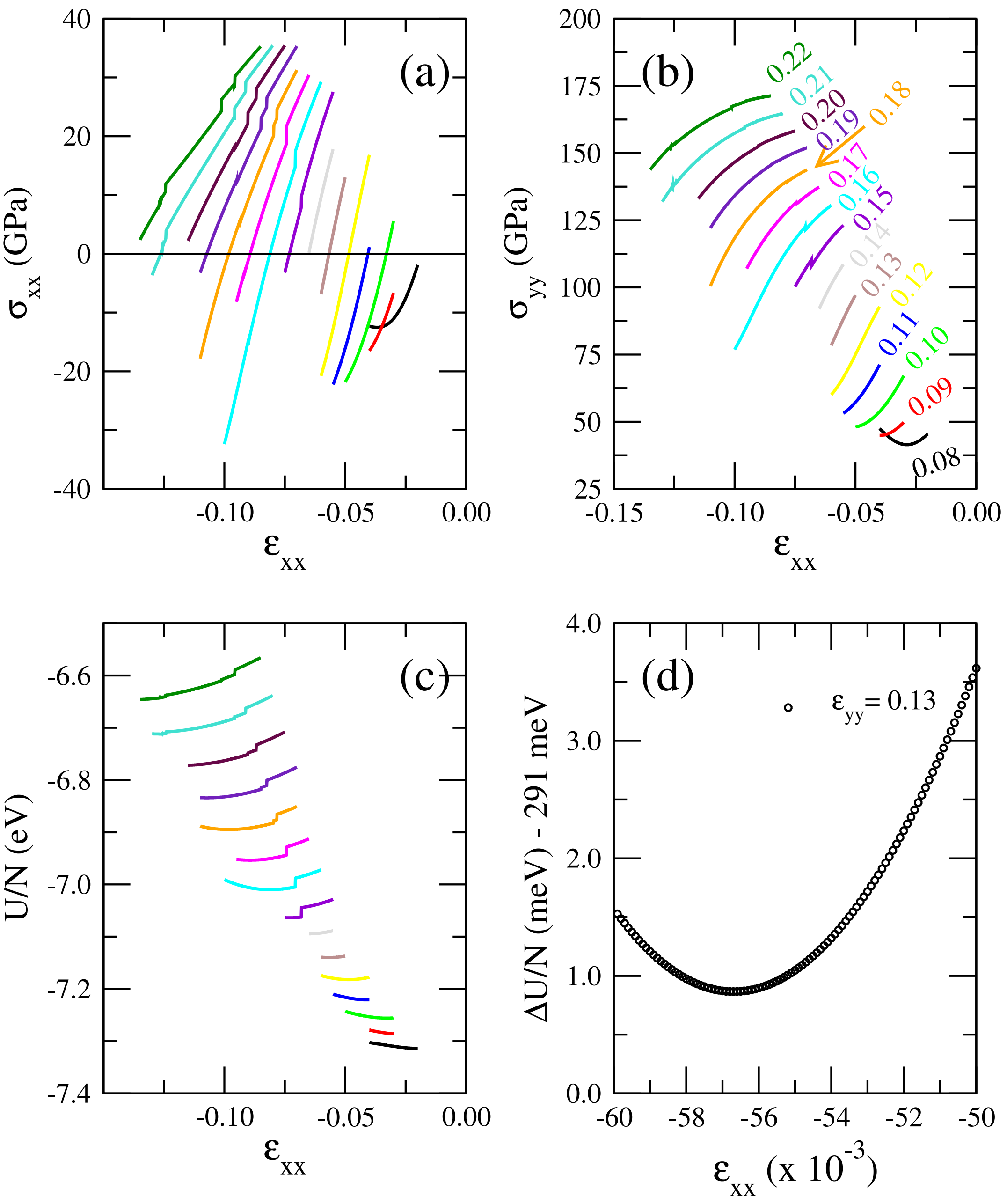

Figure S3. "Jumps" (discontinuities) in stress and energy plots obtained using the GR-RDX-2021 potential. (a) Stress along x direction (σ_{xx}), (b) stress along y direction (σ_{yy}) and (c) energy per atom (U/N) as a function of strain along x direction (ε_{xx}) for fixed ε_{yy} values indicated with different colors. The ε_{yy} values corresponding to each curve are shown next to each curves in panel (b) with the same color. (d) Strain energy per atom $\Delta U/N = U/N - U_{coh}$ shifted by 291 meV versus ε_{xx} for $\varepsilon_{yy} = 0.13$ showing the discontinuous change in its slope. Contrary to CHON-2019 and C-2013 potentials, GR-RDX-2021 does not exhibit any two energy minima for the same ε_{yy} value. The ε_{xx} increment in these plots is 10^{-4}.

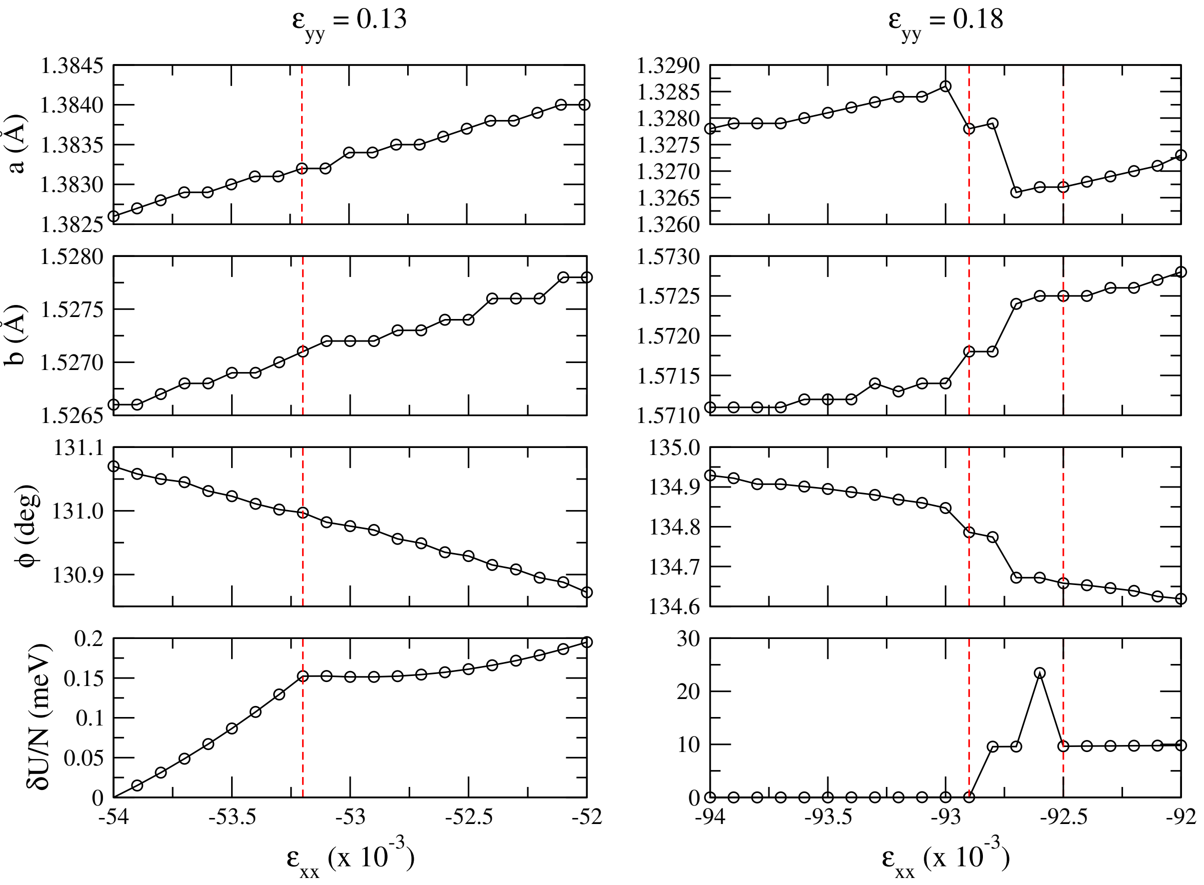

Figure S4. (Left panels) Bond lengths a and b, bond angle ϕ and energy per atom $\delta U/N$ as a function of ε_{xx} for strained graphene, obtained using the CHON-2019 ReaxFF. For the left panels, $\varepsilon_{yy} = 0.13$. For the right panels, $\varepsilon_{yy} = 0.18$. The energy plots (bottom panels) represent the energy per atom shifted by the energy value for $\varepsilon_{xx} = -0.0540$ and $\varepsilon_{yy} = 0.13$ (left panel) and by the energy value for $\varepsilon_{xx} = -0.0940$ and $\varepsilon_{yy} = 0.18$ (right panel). The dashed red lines of the left panels show the point at which the derivative $\partial U/\partial \varepsilon_{xx}$ is discontinuous. For the right panels the dashed red lines show the interval at which several discontinuities of U occur.

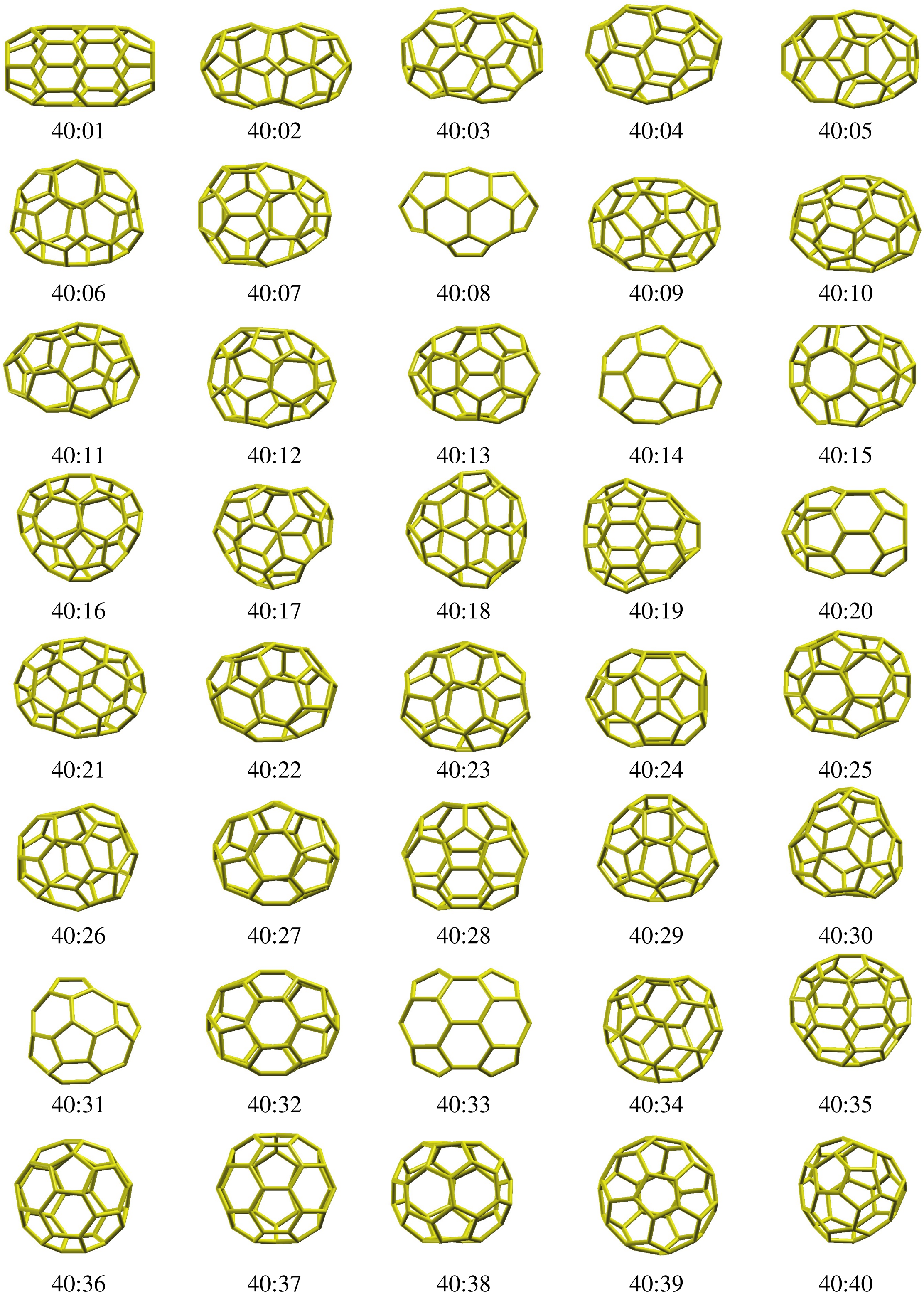

Figure S5. The optimum C_{40} fullerene isomers according to the GR-RDX-2019 potential. The isomer number according to the standard fullerene isomer enumeration is shown below each structure. The corresponding optimum structures obtained using the C-2013 and CHON-2019 potentials are very similar and topologically the same.
