# Supplementary material for: Evaluating the performance of ReaxFF potentials for sp2 carbon systems (graphene, carbon nanotubes, fullerenes) and a new ReaxFF potential
Source: Front Chem. 2022 Aug 29;10:951261. doi: 10.3389/fchem.2022.951261 (PMC9465816; doi:10.3389/fchem.2022.951261)
Supplement: Supplementary file 2 [file DataSheet1.PDF]

DATE: 2019-11-26 CONTRIBUTOR: Z. G. Fthenakis at al COMMENT: hybrid RDX and C-2013 for graphene (Cg) + C/H/O/N
39 ! Number of general parameters
50.0000 !Overcoordination parameter
9.4514 !Overcoordination parameter
30.0000 !Valency angle conjugation parameter
216.4305 !Triple bond stabilisation parameter
12.4838 !Triple bond stabilisation parameter
0.0000 !C2-correction
1.0701 !Undercoordination parameter
7.5000 !Triple bond stabilisation parameter
11.9083 !Undercoordination parameter
13.3822 !Undercoordination parameter
-10.4637 !Triple bond stabilization energy
0.0000 !Lower Taper-radius
10.0000 !Upper Taper-radius
2.8793 !Not used
33.8667 !Valency undercoordination
3.5895 !Valency angle/lone pair parameter
1.0563 !Valency angle
2.0384 !Valency angle parameter
6.1431 !Not used
6.9290 !Double bond/angle parameter
0.0283 !Double bond/angle parameter: overcoord
0.0570 !Double bond/angle parameter: overcoord
-2.4837 !Not used
5.8374 !Torsion/BO parameter
10.0000 !Torsion overcoordination
1.8820 !Torsion overcoordination
1.2327 !Conjugation 0 (not used)
2.1861 !Conjugation
1.5591 !vdWaals shielding
0.0100 !Cutoff for bond order (*100)
5.2216 !Valency angle conjugation parameter
3.4021 !Overcoordination parameter
38.5241 !Overcoordination parameter
2.1533 !Valency/lone pair parameter
0.5000 !Not used
20.0000 !Not used
5.0000 !Molecular energy (not used)
2.0000 !Version number
6.5560 !Valency angle conjugation parameter
5 ! Nr of atoms; atomID;ro(sigma); Val;atom mass;Rvdw;Dij;gamma
alfa;gamma(w);Val(angle);p(ovun5);n.u.;chiEEM;etaEEM;n.u.
ro(pipi);p(lp2);Heat increment;p(boc4);p(boc3);p(boc5),n.u.;n.u.
p(ovun2);p(val3);n.u.;Val(boc);p(val5);n.u.;n.u.;n.u.
C 1.3742 4.0000 12.0000 1.9684 0.1723 0.8712 1.2385 4.0000
9.4606 2.1346 4.0000 31.0823 79.5548 5.7254 6.9235 0.0000
1.2104 0.0000 183.7012 5.7419 33.3951 11.9957 0.8563 0.0000

```
  -2.8983   2.5000   1.0564   4.0000   2.9663   0.0000   0.0000   0.0000
H    0.6867   1.0000   1.0080   1.3525   0.0616   0.8910  -0.1000   1.0000
   9.3858   5.0013   1.0000   0.0000 121.1250   3.8446  10.0839   1.0000
  -0.1000   0.0000  58.4228   3.8461   3.2540   1.0000   1.0698   0.0000
 -15.7683   2.1504   1.0338   1.0000   2.8793   0.0000   0.0000   0.0000
O    1.3142   2.0000  15.9990   1.9741   0.0880   0.8712   1.1139   6.0000
  10.2186   7.7719   4.0000  29.5271 116.0768   8.5000   7.1412   2.0000
   0.9909  14.9473  69.2812   9.1371   1.6258   0.1863   0.9745   0.0000
  -3.5965   2.5000   1.0493   4.0000   2.9225   0.0000   0.0000   0.0000
N    1.2450   3.0000  14.0000   1.9951   0.1088   1.0512   1.1911   5.0000
   9.9303   7.8431   4.0000  32.4758 100.0000   6.7768   6.8035   2.0000
   1.0636   0.1045 128.0119   2.1604   2.9464   2.5181   0.9745   0.0000
  -4.0959   2.0047   1.0183   4.0000   2.8793   0.0000   0.0000   0.0000
Cg   1.3674 4.0000  12.0000  2.0453  0.1444 0.7920 1.1706 4.0000
   9.0000 1.5000   4.0000 27.5134 79.5548 6.7897 6.0000 0.0000
   1.1168 0.0000 181.0000 14.2732 24.4406 6.7313 0.8563 0.0000
  -4.1021 5.0000   1.0564  4.0000  2.9663 0.0000 0.0000 0.0000
15 ! Nr of bonds; at1;at2;De(sigma);De(pi);De(pipi);p(be1);p(b
p(be2);p(bo3);p(bo4);n.u.;p(bo1);p(bo2)
 1  1 141.9346 113.4487  67.6027   0.1554  -0.3045   1.0000  30.4515   0.4283
      0.0801  -0.2113   8.5395   1.0000  -0.0933   6.6967   1.0000   0.0000
 1  2 163.6889   0.0000   0.0000  -0.4525   0.0000   1.0000   6.0000   0.5921
     12.1053   1.0000   0.0000   1.0000  -0.0097   8.6351   0.0000   0.0000
 2  2 169.8421   0.0000   0.0000  -0.3591   0.0000   1.0000   6.0000   0.7503
      9.3119   1.0000   0.0000   1.0000  -0.0169   5.9406   0.0000   0.0000
 1  3 164.0476 117.4881  72.1261  -0.6031  -0.1795   1.0000  14.9755   0.5413
      1.2626  -0.3063   7.0000   1.0000  -0.1588   4.5000   0.0000   0.0000
 3  3 110.4748 155.6441  40.0000   0.1150  -0.1054   1.0000  28.5221   0.2000
      0.9590  -0.2635   8.5715   1.0000  -0.1007   6.8548   1.0000   0.0000
 1  4 130.7147 175.2276  97.2523  -0.0368  -0.4942   1.0000  26.7545   0.5133
      0.3296  -0.3653   7.0000   1.0000  -0.1171   5.1025   1.0000   0.0000
 3  4  85.4950 114.0081  70.1453   0.5778  -0.1070   1.0000  16.6611   0.2339
      0.3474  -0.1948   8.3762   1.0000  -0.1089   5.8148   1.0000   0.0000
 4  4 157.7518  67.1322 160.9732  -0.5869  -0.1824   1.0000  12.0000   0.7136
      0.8204  -0.1657  10.6490   1.0000  -0.0967   4.5976   1.0000   0.0000
 2  3 224.3076   0.0000   0.0000  -0.6280   0.0000   1.0000   6.0000   1.0000
      5.0050   1.0000   0.0000   1.0000  -0.0512   5.1982   0.0000   0.0000
 2  4 212.1772   0.0000   0.0000  -0.3585   0.0000   1.0000   6.0000   0.3316
     10.4316   1.0000   0.0000   1.0000  -0.0658   6.4545   0.0000   0.0000
1 5 141.9346 113.4487  67.6027   0.1554  -0.3045   1.0000  30.4515   0.4283
      0.0801  -0.2113   8.5395   1.0000  -0.0933   6.6967   1.0000   0.0000
2 5 163.6889   0.0000   0.0000  -0.4525   0.0000   1.0000   6.0000   0.5921
     12.1053   1.0000   0.0000   1.0000  -0.0097   8.6351   0.0000   0.0000
3 5 164.0476 117.4881  72.1261  -0.6031  -0.1795   1.0000  14.9755   0.5413
      1.2626  -0.3063   7.0000   1.0000  -0.1588   4.5000   0.0000   0.0000
4 5 130.7147 175.2276  97.2523  -0.0368  -0.4942   1.0000  26.7545   0.5133
      0.3296  -0.3653   7.0000   1.0000  -0.1171   5.1025   1.0000   0.0000
5 5 80.8865 107.9944 52.0636 0.5218 -0.3636 1.0000 34.9876 0.7769
   6.1244  -0.1693  8.0804 1.0000 -0.0586 8.1850  1.0000 0.0000
```

9 ! Nr of off-diagonal terms. at1;at2;Dij;RvdW;alfa;ro(sigma);r
 1 2   0.0464   1.8296 10.1311   1.0029 -1.0000 -1.0000
 2 3   0.0375   1.7275 10.8037   0.8813 -1.0000 -1.0000
 2 4   0.0509   1.7672 10.4261   0.9990 -1.0000 -1.0000
 1 3   0.1036   1.8869   9.5668   1.3590   1.1099   1.1534
 1 4   0.1971   1.7356 10.0734   1.2754   1.2113   1.1172
 3 4   0.0535   1.6709 10.8180   1.2968   1.1416   1.0167
2 5   0.0464   1.8296 10.1311   1.0029 -1.0000 -1.0000
3 5   0.1036   1.8869   9.5668   1.3590   1.1099   1.1534
4 5   0.1971   1.7356 10.0734   1.2754   1.2113   1.1172
75 ! Nr of angles. at1;at2;at3;Theta0_0;ka;kb;pv1;pv2;kpenal;pv3
 1 1 1 74.0317 32.2712   0.9501   0.0000   0.1780 10.5736   1.0400
 1 1 2 70.6558 14.3658   5.3224   0.0000   0.0058   0.0000   1.0400
 1 1 3 65.3104   6.3897   7.5000   0.0000   0.2000 10.0000   1.8525
 1 1 4 65.8892 45.0000   1.6598   0.0000   0.2000 10.0000   1.8525
 1 1 5 74.0317 32.2712   0.9501   0.0000   0.1780 10.5736   1.0400
 2 1 2 76.7339 14.4217   3.3631   0.0000   0.0127   0.0000   1.0400
 2 1 3 56.3039 17.3681   5.3095   0.0000   0.9110   0.0000   1.0400
 2 1 4 71.5505 11.1820   3.7129   0.0000   0.9110   0.0000   1.0400
 2 1 5 70.6558 14.3658   5.3224   0.0000   0.0058   0.0000   1.0400
 3 1 3 71.9855 28.5708   6.4252   0.0000   0.2000   0.0000   1.8525
 3 1 4 73.1057 25.8227   4.2145   0.0000   0.2000   0.0000   1.8525
 3 1 5 65.3104   6.3897   7.5000   0.0000   0.2000 10.0000   1.8525
 4 1 4 65.8759 40.9838   2.4369   0.0000   0.2000   0.0000   1.8525
 4 1 5 65.8892 45.0000   1.6598   0.0000   0.2000 10.0000   1.8525
 5 1 5 74.0317 32.2712   0.9501   0.0000   0.1780 10.5736   1.0400
 1 2 1   0.0000   3.4110   7.7350   0.0000   0.0000   0.0000   1.0400
 1 2 2   0.0000   0.0000   6.0000   0.0000   0.0000   0.0000   1.0400
 1 2 3   0.0000   0.0019   6.0000   0.0000   0.0000   0.0000   1.0400
 1 2 4   0.0000   0.0019   6.0000   0.0000   0.0000   0.0000   1.0400
 1 2 5   0.0000   3.4110   7.7350   0.0000   0.0000   0.0000   1.0400
 2 2 2   0.0000 27.9213   5.8635   0.0000   0.0000   0.0000   1.0400
 2 2 3   0.0000   0.0019   6.0000   0.0000   0.0000   0.0000   1.0400
 2 2 4   0.0000   0.0019   6.0000   0.0000   0.0000   0.0000   1.0400
 2 2 5   0.0000   0.0000   6.0000   0.0000   0.0000   0.0000   1.0400
 3 2 3   0.0000   0.0019   6.0000   0.0000   0.0000   0.0000   1.0400
 3 2 4   0.0000   0.0019   6.0000   0.0000   0.0000   0.0000   1.0400
 3 2 5   0.0000   0.0019   6.0000   0.0000   0.0000   0.0000   1.0400
 4 2 4   0.0000   0.0019   6.0000   0.0000   0.0000   0.0000   1.0400
 4 2 5   0.0000   0.0019   6.0000   0.0000   0.0000   0.0000   1.0400
 5 2 5   0.0000   3.4110   7.7350   0.0000   0.0000   0.0000   1.0400
 1 3 1 72.3642 37.8942   1.1566   0.0000   0.7472   0.0000   1.2639
 1 3 2 89.1394 37.0874   0.3849   0.0000   3.0000   0.0000   1.2618
 1 3 3 90.0000 45.0000   0.5719   0.0000   0.7472   0.0000   1.2639
 1 3 4 70.4313 14.4055   7.1593   0.0000   0.7472   0.0000   1.2639
 1 3 5 72.3642 37.8942   1.1566   0.0000   0.7472   0.0000   1.2639
 2 3 2 82.3474 13.5165   3.4896   0.0000   0.3596   0.0000   1.3307
 2 3 3 80.7068   5.0854   5.7151   0.0000   3.0000   0.0000   1.2618
 2 3 4 76.0238 45.0000   0.8637   0.0000   3.0000   0.0000   1.2618

2	3	5	89.1394	37.0874	0.3849	0.0000	3.0000	0.0000	1.2618
3	3	3	83.8833	23.3345	2.3433	-10.0000	0.7472	0.0000	1.2639
3	3	4	84.0407	45.0000	1.0695	0.0000	0.7472	0.0000	1.2639
3	3	5	90.0000	45.0000	0.5719	0.0000	0.7472	0.0000	1.2639
4	3	4	73.9966	24.4410	5.2760	0.0000	0.7472	0.0000	1.2639
4	3	5	70.4313	14.4055	7.1593	0.0000	0.7472	0.0000	1.2639
5	3	5	72.3642	37.8942	1.1566	0.0000	0.7472	0.0000	1.2639
1	4	1	68.4330	19.3525	2.1625	0.0000	1.7325	0.0000	1.0440
1	4	2	90.0000	32.0540	0.7195	0.0000	0.5355	0.0000	2.5279
1	4	3	86.2893	37.5587	1.2660	0.0000	1.7325	0.0000	1.0440
1	4	4	74.2404	12.0547	7.5000	0.0000	1.7325	0.0000	1.0440
1	4	5	68.4330	19.3525	2.1625	0.0000	1.7325	0.0000	1.0440
2	4	2	56.3036	14.1532	3.3914	0.0000	0.2000	0.0000	2.1689
2	4	3	84.1185	45.0000	1.3826	0.0000	0.5355	0.0000	2.5279
2	4	4	78.7133	24.6250	3.8202	0.0000	0.5355	0.0000	2.5279
2	4	5	90.0000	32.0540	0.7195	0.0000	0.5355	0.0000	2.5279
3	4	3	78.5566	43.8492	1.3351	-26.1471	1.7325	40.0000	1.0440
3	4	4	77.4239	33.7297	1.7944	-0.9193	1.7325	0.0000	1.0440
3	4	5	86.2893	37.5587	1.2660	0.0000	1.7325	0.0000	1.0440
4	4	4	64.9107	17.5558	7.5000	0.0000	1.7325	0.0000	1.0440
4	4	5	74.2404	12.0547	7.5000	0.0000	1.7325	0.0000	1.0440
5	4	5	68.4330	19.3525	2.1625	0.0000	1.7325	0.0000	1.0440
1	5	1	74.0317	32.2712	0.9501	0.0000	0.1780	10.5736	1.0400
1	5	2	70.6558	14.3658	5.3224	0.0000	0.0058	0.0000	1.0400
1	5	3	65.3104	6.3897	7.5000	0.0000	0.2000	10.0000	1.8525
1	5	4	65.8892	45.0000	1.6598	0.0000	0.2000	10.0000	1.8525
1	5	5	74.0317	32.2712	0.9501	0.0000	0.1780	10.5736	1.0400
2	5	2	76.7339	14.4217	3.3631	0.0000	0.0127	0.0000	1.0400
2	5	3	56.3039	17.3681	5.3095	0.0000	0.9110	0.0000	1.0400
2	5	4	71.5505	11.1820	3.7129	0.0000	0.9110	0.0000	1.0400
2	5	5	70.6558	14.3658	5.3224	0.0000	0.0058	0.0000	1.0400
3	5	3	71.9855	28.5708	6.4252	0.0000	0.2000	0.0000	1.8525
3	5	4	73.1057	25.8227	4.2145	0.0000	0.2000	0.0000	1.8525
3	5	5	65.3104	6.3897	7.5000	0.0000	0.2000	10.0000	1.8525
4	5	4	65.8759	40.9838	2.4369	0.0000	0.2000	0.0000	1.8525
4	5	5	65.8892	45.0000	1.6598	0.0000	0.2000	10.0000	1.8525

5 5 5 74.9085 44.7514 0.9144 0.0000 0.0050 0.3556 2.5715

44 ! Nr of torsions. at1;at2;at3;at4;;V1;V2;V3;p(tor1);p(cot1);n

1	1	1	1	0.0000	48.4194	0.3163	-8.6506	-1.7255	0.0000	0.0000
1	1	1	2	0.0000	63.3484	0.2210	-8.8401	-1.8081	0.0000	0.0000
1	1	1	5	0.0000	48.4194	0.3163	-8.6506	-1.7255	0.0000	0.0000
5	1	1	5	0.0000	48.4194	0.3163	-8.6506	-1.7255	0.0000	0.0000
2	1	1	2	0.0000	45.2741	0.4171	-6.9800	-1.2359	0.0000	0.0000
2	1	1	5	0.0000	63.3484	0.2210	-8.8401	-1.8081	0.0000	0.0000
0	1	1	0	0.0930	18.5962	0.0002	-9.0000	-1.0000	0.0000	0.0000
0	1	2	0	0.0000	0.0000	0.0000	0.0000	0.0000	0.0000	0.0000
1	1	3	3	-0.0002	21.5452	0.1727	-9.0000	-2.0000	0.0000	0.0000
3	1	3	3	-1.3476	22.4932	1.5000	-9.0000	-2.0000	0.0000	0.0000
3	1	3	5	-0.0002	21.5452	0.1727	-9.0000	-2.0000	0.0000	0.0000

```
  0  1  3  0  -0.0002  85.8794   0.3236  -3.8134  -2.0000   0.0000   0.0000
  4  1  4  4  -2.0000  20.8732  -1.5000  -9.0000  -2.0000   0.0000   0.0000
  0  1  4  0  -0.0069 150.0000   0.4891  -7.4921  -2.0000   0.0000   0.0000
  1  1  5  1   0.0000  48.4194   0.3163  -8.6506  -1.7255   0.0000   0.0000
  1  1  5  2   0.0000  63.3484   0.2210  -8.8401  -1.8081   0.0000   0.0000
  1  1  5  5   0.0000  48.4194   0.3163  -8.6506  -1.7255   0.0000   0.0000
  2  1  5  2   0.0000  45.2741   0.4171  -6.9800  -1.2359   0.0000   0.0000
  2  1  5  5   0.0000  63.3484   0.2210  -8.8401  -1.8081   0.0000   0.0000
  5  1  5  5   0.0000  48.4194   0.3163  -8.6506  -1.7255   0.0000   0.0000
  0  1  5  0   0.0930  18.5962   0.0002  -9.0000  -1.0000   0.0000   0.0000
  0  2  2  0   0.0000   0.0000   0.0000   0.0000   0.0000   0.0000   0.0000
  0  2  3  0   0.0000   0.1000   0.0200  -2.5415   0.0000   0.0000   0.0000
  0  2  4  0   0.0000   0.1000   0.0200  -2.5415   0.0000   0.0000   0.0000
  0  2  5  0   0.0000   0.0000   0.0000   0.0000   0.0000   0.0000   0.0000
  1  3  3  1   0.0002  79.3777  -1.5000  -5.2139  -2.0000   0.0000   0.0000
  1  3  3  5   0.0002  79.3777  -1.5000  -5.2139  -2.0000   0.0000   0.0000
  5  3  3  5   0.0002  79.3777  -1.5000  -5.2139  -2.0000   0.0000   0.0000
  0  3  3  0  -0.9667 116.4743   0.0002  -4.9422   0.0000   0.0000   0.0000
  0  3  4  0   1.6745  56.6301  -0.0008  -4.5064  -2.0000   0.0000   0.0000
  1  3  5  3  -0.0002  21.5452   0.1727  -9.0000  -2.0000   0.0000   0.0000
  3  3  5  3  -1.3476  22.4932   1.5000  -9.0000  -2.0000   0.0000   0.0000
  3  3  5  5  -0.0002  21.5452   0.1727  -9.0000  -2.0000   0.0000   0.0000
  0  3  5  0  -0.0002  85.8794   0.3236  -3.8134  -2.0000   0.0000   0.0000
  0  4  4  0   1.1253  75.3447   0.0080  -9.0000  -2.0000   0.0000   0.0000
  4  4  5  4  -2.0000  20.8732  -1.5000  -9.0000  -2.0000   0.0000   0.0000
  0  4  5  0  -0.0069 150.0000   0.4891  -7.4921  -2.0000   0.0000   0.0000
  1  5  5  1   0.0000  48.4194   0.3163  -8.6506  -1.7255   0.0000   0.0000
  1  5  5  2   0.0000  63.3484   0.2210  -8.8401  -1.8081   0.0000   0.0000
  1  5  5  5   0.0000  48.4194   0.3163  -8.6506  -1.7255   0.0000   0.0000
  2  5  5  2   0.0000  45.2741   0.4171  -6.9800  -1.2359   0.0000   0.0000
  2  5  5  5   0.0000  63.3484   0.2210  -8.8401  -1.8081   0.0000   0.0000
  0  5  5  0   0.0930  18.5962   0.0002  -9.0000  -1.0000   0.0000   0.0000
5 5 5 5 2.1207 26.8713 0.5160 -9.0000 -2.8394 0.0000 0.0000
4 ! Nr of hydrogen bonds. at1;at2;at3;r(hb);p(hb1);p(hb2);p(hb3
  3  2  3   2.0000  -5.0000   3.0000   3.0000
  3  2  4   1.7753  -5.0000   3.0000   3.0000
  4  2  3   1.3884  -5.0000   3.0000   3.0000
  4  2  4   1.6953  -4.0695   3.0000   3.0000
```
